# Judicious Use of Chalcogens in Multiresonant Thermally Activated Delayed Fluorescent Emitters Leads to OLEDs with Efficiencies Exceeding 36% and Showing Mild Efficiency Roll‐Off

**DOI:** 10.1002/anie.202511866

**Published:** 2025-08-06

**Authors:** Futong Liu, Zihan Su, Zhuang Cheng, Dongyang Chen, Yangze Xu, Yan Yan, Wei Dong, Liang Wan, Eli Zysman‐Colman, Ping Lu

**Affiliations:** ^1^ State Key Laboratory of Supramolecular Structure and Materials Department of Chemistry Jilin University Changchun 130012 P.R. China; ^2^ Organic Semiconductor Centre EaStCHEM School of Chemistry University of St Andrews St Andrews KY16 9ST UK

**Keywords:** Heavy atom effect, Multi‐resonance thermally activated delayed fluorescence, Narrowband emission, Reverse intersystem crossing, Small efficiency roll‐off

## Abstract

Strategies that produce OLEDs that simultaneously achieve outstanding electroluminescence efficiency, small efficiency roll‐off, good stability, and high color purity remain in strong demand. Herein, we report how judicious decoration of a benzochalcogenophene onto the **BCzBN** core leads to an acceleration of reverse intersystem crossing (*k*_RISC_) without sacrificing either color fidelity and photoluminescence quantum yield (*Φ*_PL_). Compared with **BCzBN**, the *k*_RISC_ of **BN‐S** and **BN‐Se** are accelerated to 2.5 × 10^5^ and 7.2 × 10^5^ s^−1^ from 1.4 × 10^4^ s^−1^, each with near‐unity *Φ*_PL_s. **BN‐S** and **BN‐Se** show narrowband emission at 483 nm (FWHM of 22 nm) and 485 nm (FWHM of 22 nm). The corresponding non‐sensitized OLEDs using **BN‐S** and **BN‐Se** exhibited record‐breaking maximum external quantum efficiency (EQE_max_) of 43.1 and 36.9%, without any external light extraction techniques. Furthermore, the **BN‐Se**‐based OLED showed ultralow efficiency roll‐off, with EQEs remaining at 36.7 and 31.8% at 100 and 1000 cd m^−2^, respectively. These emitters do not easily suffer from aggregation‐caused quenching. Even at 12 wt% doping, the EQE_max_ of the OLEDs with **BN‐S** and **BN‐Se** were 36.7 and 36.4%, respectively, with mild efficiency roll‐off (EQEs at 1000 cd m^−2^ of 19.1 and 30.8%, respectively), and nearly unchanged electroluminescence spectra.

## Graphical abstract

At doping ratios ranging from 1 to 12 wt%, the **BN‐S** and **BN‐Se**‐based sensitizer‐free OLEDs exhibited nearly unchanged EL spectra with small FWHMs of 27–30 nm and EQE_max_ of 36.4–43.1%. The **BN‐Se**‐based OLED exhibited low efficiency roll‐off, with EQEs remaining at 36.7 and 31.8% at 100 and 1000 cd m^−2^, respectively, representing the best EL performance among mono‐boron MR‐TADF OLEDs to date.

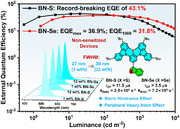

## Introduction

Thermally activated delayed fluorescence (TADF) emitters have received widespread attention owing to their capacity to harvest 100% of the electrically generated excitons and convert them into light, mediated by reverse intersystem crossing (RISC) of dark triplets to bright singlets.^[^ ^1^, ^2^, ^3^ ^]^ The conventional design strategy for TADF materials involves the construction of a highly twisted donor‐acceptor (D‐A) structure, which sufficiently separates the HOMO and LUMO to induce a small singlet‐triplet energy gap (Δ*E*_ST_) and facilitates RISC.^[^ ^4^, ^5^, ^6^, ^7^ ^]^ However, such designs lead to emissive excited states possessing long‐range charge‐transfer (LRCT) character, which typically results in significant structural relaxation of the S_1_ state, leading to broad emission spectra evidenced by full‐width at half maxima (FWHM) typically larger than 70 nm. These emitters thus cannot match the strict BT.2020 industry requirements for the blue color gamut for ultra‐high‐definition displays.

A potential solution to this long‐standing bottleneck was reported in 2016 when Hatakeyama and co‐workers introduced multi‐resonance TADF (MR‐TADF) emitters.^[^ ^8^ ^]^ These compounds are p‐ and n‐doped polycyclic aromatic hydrocarbon frameworks, in which the precise regiochemistry of these dopants (N and B, in the case of this report of **DABNA‐1** and **DABNA‐2**) produces a separation of the frontier molecular orbitals that leads to low‐lying short‐range charge‐transfer (SRCT) excited states and sufficiently small Δ*E*_ST_ to enable TADF. Moreover, their rigid structure suppresses structural relaxation in the excited state, resulting in small Stokes shifts and narrowband emission (FWHMs < 50 nm).^[^ ^9^, ^10^ ^]^ To date, numerous high‐performance MR‐TADF materials covering the entire color gamut have been developed.^[^ ^11^, ^12^, ^13^, ^14^, ^15^, ^16^, ^17^, ^18^, ^19^ ^]^ A long‐lasting issue, though, is that most MR‐TADF emitters, because of their moderately large Δ*E*_ST_ values, have relatively slow TADF kinetics due to a too slow RISC rate constant (*k*_RISC_) in the range of 10^3^–10^4^ s^−1^.^[^ ^20^, ^21^, ^22^, ^23^ ^]^ This leads to rather severe efficiency roll‐off in the OLEDs at high luminance due to triplet exciton‐involved quenching processes.^[^ ^24^, ^25^, ^26^, ^27^ ^]^

The magnitude of *k*_RISC_ is mainly controlled by the Δ*E*_ST_ and the spin‐orbit coupling (SOC) matrix element between the S_1_ and T_1_.^[^ ^28^, ^29^, ^30^ ^]^ One solution that has received intense attention recently is to increase SOC by integrating heavy elements into the MR‐TADF skeleton as SOC scales as *Z* ^4^.^[^ ^31^, ^32^, ^33^, ^34^, ^35^, ^36^, ^37^, ^38^, ^39^, ^40^, ^41^, ^42^, ^43^ ^]^ For instance, Yasuda and co‐workers reported a selenium‐doped organoboron emitter **CzBSe**, which has a *k*_RISC_ of 1.8 × 10^8^ s^−1^ (in 1 wt% doped films in mCBP), a high EQE_max_ of 23.9% and an EQE_1000_ of 20.0%, showing a low efficiency roll‐off of 16% at 1000 cd m^−2^.^[^ ^34^ ^]^ Yang and co‐workers prepared a series of MR‐TADF molecules by integrating a phenoselenazine (**PSeZ**) moiety into the polycyclic BN‐scaffold, and of these, **BNSeSe**‐based OLEDs demonstrated a high EQE_max_ of 36.8% having a small efficiency roll‐off (EQE_1000_/EQE_10000_: 34.0/26.0%).^[^ ^35^ ^]^ Zysman–Colman and co‐workers identified the optimal regiochemistry of a sulfur‐containing benzothiophenocarbazole moiety in MR‐TADF emitters for maximizing SOC and accelerating RISC. **tCzBT1B** and **tCzBT2B** have *k*_RISC_ of 1.2 × 10^5^ and 2.7 × 10^5^ s^−1^, respectively, which translated into sensitizer‐free OLEDs with **tCzBT1B** and **tCzBT2B** showing high EQE_max_ of 34.9% and 34.3% and mild efficiency roll‐off (EQE_1000_: 18.0/22.4%).^[^ ^36^ ^]^ Yang and co‐workers reported a peripheral selenoxanthone‐incorporated MR‐TADF emitter **BN‐STO** where the device showed an outstanding EQE_max_ of 40.1%, significant efficiency roll‐off (EQE_1000_ of 28.1%).^[^ ^37^ ^]^ Su and co‐workers reported two MR‐TADF emitters, **pPXSe‐BN** and **mPXSe‐BN**, that contain embedded phenoxaselenines within the MR‐TADF structure at different substituted positions. The device with **pPXSe‐BN** achieved a high EQE_max_ of 31.7% and an EQE_1000_ of 26.7% using the exciplex cohost.^[^ ^38^ ^]^ Our group also designed a sulfur‐fusing asymmetric MR‐TADF molecule **2Cz‐PTZ‐BN**, which exhibited a fast *k*_RISC_ above 1.0 × 10^5^ s^−1^ (in 3 wt% doped films in PhCzBCz), a high EQE_max_ of 32.8% and maintained an EQE of 23.5% at 1000 cd m^−2^.^[^ ^39^ ^]^ Although there has been success in accelerating *k*_RISC_ using this strategy, the direct embedding of sulfur or selenium atoms in MR‐TADF frameworks is not an ideal choice because of the tradeoffs of larger FWHM and multi‐step synthesis to integrate the chalcogen atoms.^[^ ^44^, ^45^ ^]^ Therefore, a simple and effective strategy to synchronously improve the efficiency (EQE > 40%), increase the RISC rate (k_RISC_ > 10^5^ s^−1^), and conserve color purity (FWHM < 30 nm) without requiring a costly multi‐step synthesis remains an outstanding challenge for emitter development.

**Figure 1:**
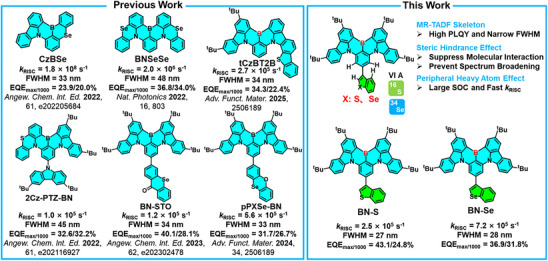
Molecular design and chemical structures of MR‐TADF emitters.

Herein, we designed two MR‐TADF molecules **BN‐S** and **BN‐Se** by attaching thianaphthene and benzo[b]selenophene as peripheral groups at the *para* positions of the B‐substituted phenyl ring (Figure 1) of a **tCzBN** core.^[^ ^46^, ^47^ ^]^ Embedding the sulfur/selenium in the chalcogenophenes addresses an issue of photochemical and thermal stability by tethering the heavy atom‐containing group to the MR‐TADF core as opposed to integrating it within the PAH where weak C─S or C─Se single bonds are points of scission.^[^ ^40^, ^41^ ^]^ The sulfur and selenium atoms from thiophene and selenophene not only are electron‐donating, but also enhance SOC and thus accelerate RISC.^[^ ^48^, ^49^ ^]^ As a result, **BN‐S** and **BN‐Se** exhibit almost identical narrowband emissions peaking at 483 and 485 nm, respectively, with the same FWHM values of 22 nm. In comparison to parent molecule **BCzBN**, **BN‐S** and **BN‐Se** have larger SOC, reflected in their shorter delayed fluorescence lifetimes, *τ*_d_, of 11.5 and 3.5 µs and faster *k*_RISC_ values of 2.5 × 10^5^ and 7.2 × 10^5^ s^−1^. Consequently, without employing an additional sensitizer, the optimized OLEDs using **BN‐S** exhibit an outstandingly high maximum EQE of 43.1% and a narrow FWHM of 27 nm, while the devices with **BN‐Se** showed a lower EQE_max_ of 36.9% (FWHM of 28 nm). The performance of the **BN‐S** device places it amongst the most efficient to date MR‐TADF OLEDs. At doping ratios ranging from 1 to 12 wt%, the **BN‐S** and **BN‐Se**‐based OLEDs exhibit nearly unchanged electroluminescence (EL) spectra and remarkably conserved high EQE_max_ values of 36.7%–43.1% and 36.4%–36.9%, respectively. It is noteworthy that the **BN‐Se**‐based device shows impressively mild efficiency roll‐off with the EQE remaining at 36.7% and 31.8% at luminances of 100 and 1000 cd m^−2^, respectively.

## Results and Discussion

### Synthesis and Characterization

The synthesis routes to **BN‐S** and **BN‐Se** are shown in Scheme S1. The precursors **SeBr** and **DtCzB‐Bpin** were prepared in high yields following literature procedures,^[^ ^48^, ^50^ ^]^ and the target emitters were synthesized following a one‐step Pd‐catalyzed Suzuki–Miyaura cross‐coupling reaction. The molecular structures of **BN‐S** and **BN‐Se** were confirmed by NMR spectroscopy, mass spectrometry, and elemental analysis (Figures S1–S8). **BN‐S** and **BN‐Se** showed high decomposition temperatures (*T*_d_, 5 wt% loss) of 443 and 440 °C, respectively, by thermal gravimetric analysis (Figure S9a). During the second heating cycle of the differential scanning calorimetry (DSC) traces, no obvious exo‐ or endothermic signals were observed from 100 to 350 °C, which indicates melting temperatures above 350 °C. The energies of the HOMO and LUMO were inferred from the cyclic voltammetry (CV) measurements, where the oxidation and reduction potentials (*E*_ox_/*E*_red_) are reported against ferrocenium/ferrocene (Fc^+^/Fc) redox couple as the internal standard (Figure S9b). The HOMO energy levels were estimated from the onsets of the oxidation potentials (*E*_ox_ of 0.88 for **BN‐S** and 0.90 V for **BN‐Se**) to be approximately −5.46 and −5.48 eV for **BN‐S** and **BN‐Se**, while the corresponding LUMO energy levels were estimated from the onset of the reduction potentials (*E*_red_ of −1.88 and −1.85 V for **BN‐S** and **BN‐Se**) to be −2.85 and −2.88 eV, respectively.

### Single‐Crystal Structures

**Figure 2:**
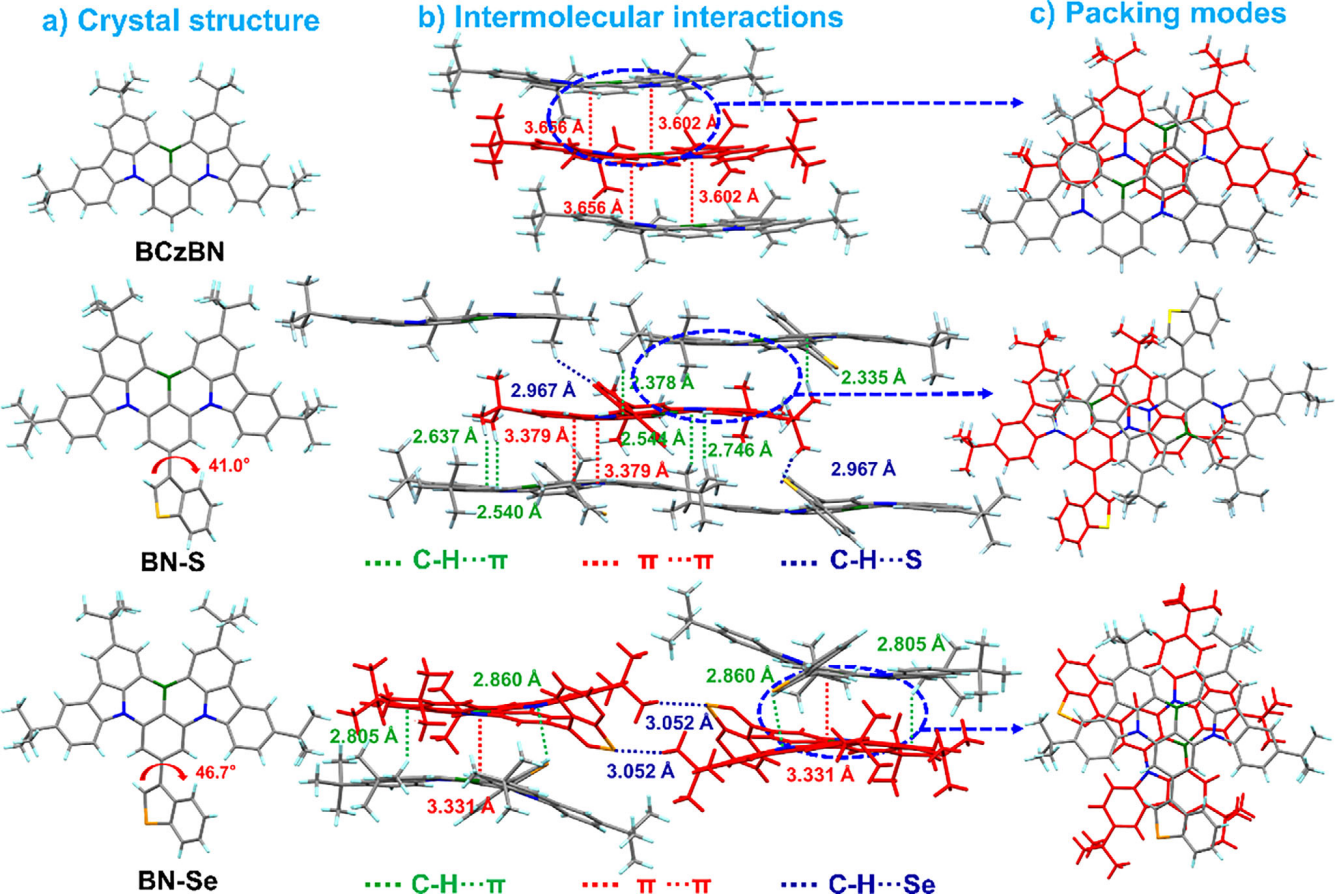
Single‐crystal structures, intermolecular interactions and packing modes of **BCzBN** (CCDC 2074929), **BN‐S** (CCDC 2381597), and **BN‐Se** (CCDC 2472002) in crystals.

Single‐crystals of **BN‐S** were obtained during gradient temperature sublimation, while those of **BN‐Se** were obtained by slow diffusion of ethanol into a saturated solution of the emitter in dichloromethane over several days. As depicted in Figure 2, the structures of **BN‐S** and **BN‐Se** adopt twisted geometries, with moderate torsion angles of 41.0° and 46.7° between the **tCzBN**‐core and the adjacent thianaphthene and benzo[b]selenophene moieties, respectively, which we hypothesize will provide some intermolecular steric repulsion to suppress undesired aggregation‐caused quenching in the solid state. In terms of molecular packing, only head‐to‐head intermolecular interactions were observed between adjacent **BCzBN** moieties, with intermolecular distances of 3.55–3.66 Å. These close contacts are likely responsible for the observed red‐shifted emission and spectral broadening in the film at 12 wt% doping concentration (Figure S22).^[^ ^51^ ^]^ Distinct from the stacking mode of **BCzBN**, **BN‐S** and **BN‐Se** stack in a head‐to‐tail pattern reinforced by multiple supramolecular interactions, such as strong π⋯π stacking with inter‐planar spacing of 3.331–3.379 Å, multiple non‐covalent C─H⋯π interactions (2.335–2.860 Å), and apparently strong intermolecular H‐bonds (2.967–3.052 Å) between the sulfur/selenium atoms in the thianaphthene or benzo[b]selenophene groups and the hydrogen atom of **BCzBN**.

### Theoretical Calculations

**Figure 3:**
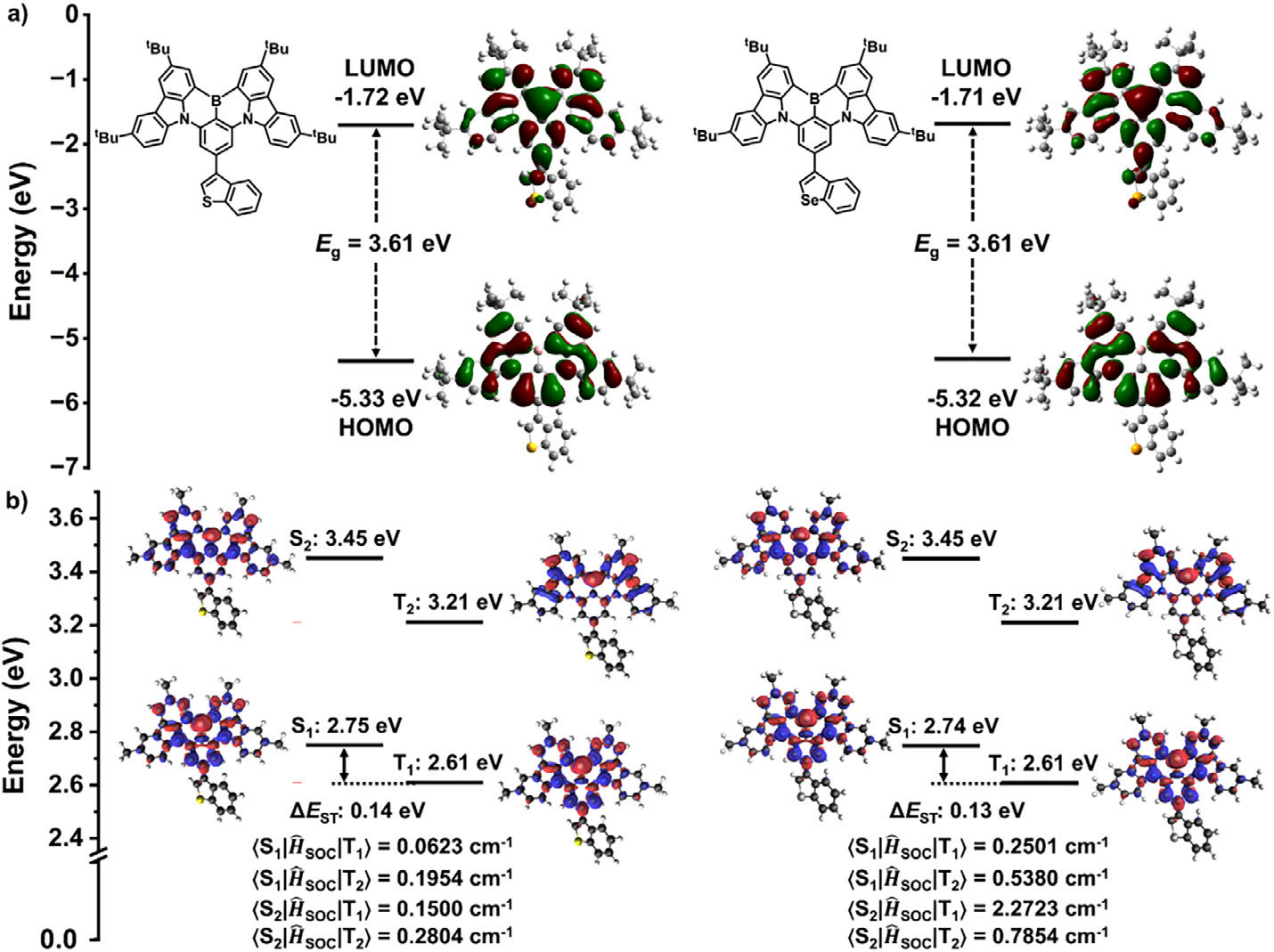
a) HOMO and LUMO electron density distribution and orbital energies of **BN‐S** and **BN‐Se** calculated at PBE0/6–31G(d,p) level in the gas phase; b) The natural transition orbitals (NTOs) and energies of S_1_, T_1_, S_2_, and T_2_ as well as spin‐orbit coupling (SOC) matrix elements for **BN‐S** and **BN‐Se** calculated at SCS‐ADC2/cc‐pVDZ level in the gas phase.

To gain insight into the molecular geometry and electronic property, quantum chemical calculations were performed at PBE0/6–31G(d,p) level. As illustrated in Figure S10, the optimized ground state (S_0_) and S_1_ geometries of **BN‐S** and **BN‐Se** are similar, indicating that there is very small structural relaxation upon electronic excitation. The thianaphthene and benzo[b]selenophene units are twisted out of the plane of the **tCzBN** core, with dihedral angles of 45.5°–50.9° in both the S_0_ and S_1_ geometries. These twisted conformations predicted by DFT calculations are consistent with the single crystal X‐ray diffraction analysis (Figure S11), indicating that this level of theory accurately predicts the ground‐state geometries of MR‐TADF systems. The calculated reorganization energies (*λ*) were estimated to be 0.1029 eV for **BN‐S** and 0.1037 eV for **BN‐Se**, respectively, which are smaller than **BCzBN** (0.1238 eV).^[^ ^51^ ^]^ This is because the vibrational relaxations and structural changes were significantly suppressed in the former two, which should lead to a more narrowband emission, reduced non‐radiative decay rate, and enhanced RISC. The HOMO and LUMO of **BN‐S** and **BN‐Se** reside primarily on the **BCzBN** moiety (Figure 3a) and have nearly identical HOMO/LUMO energy levels between the two emitters. The trends in the orbital energies align well with the CV measurements (Figure S9b). Further, the high oscillator strength (*f*) values of 0.5580 and 0.5578 for the S_0_–S_1_ transition should translate to high *Φ*_PL_. From the electrostatic potential (ESP) analysis (Figure S12), the introduction of benzochalcogenophene units at the *para*‐carbon position of the B‐containing aryl ring expands the ESP distribution to varying degrees as a function of the chalcogen. To qualitatively assess the through‐space intramolecular noncovalent interactions, we performed reduced density gradient (RDG) analysis (Figures S13 and S14). The isosurfaces are colored based on sign(*λ*_2_)*ρ* (the amplitude of the electron density corresponding to different types of interactions), where blue and green regions indicate strong interactive hydrogen bonds and van der Waals interactions, and red regions indicate areas where there are repulsive interactions, such as steric hindrance interactions. The peripheral benzochalcogenophenes and **tCzBN** moiety are colored green, indicating that there are relatively strong intramolecular interactions, which helps to explain the conformation adopted by the benzochalcogenophene. The RDG scattering diagrams also show several spikes in the low‐gradient region of the sign(*λ*_2_)*ρ* function, indicating that there are strong intramolecular noncovalent van der Waals interactions in both **BN‐S** and **BN‐Se**. These structural features are favorable for suppressing aggregation‐caused quenching and preserving the strong solid‐state luminescence. To gain deeper insight into the photochemical and thermal stabilities of **BN‐Se** and **BN‐Se** at the molecular level, key bond dissociation energies (BDE) associated were calculated based on the optimized S_0_ geometry (Figure S15). The calculated BDE results suggest that the C─S or C─Se single bonds in model compounds **BN‐SPh** (71.98 kcal mol^−1^) and **BN‐SePh** (80.82 kcal mol^−1^) are less stable than the C─C bonded structures of **BN‐S** (116.96 kcal mol^−1^) and **BN‐Se** (114.99 kcal mol^−1^). This implies that these latter two compounds are more thermally and photochemically stable than the model compounds and that the incorporation of the heavy chalcogens within a C‐bonded benzochalcogenophene is an advantageous element in MR‐TADF emitter design.

Natural transition orbital (NTO) analysis was performed to investigate the excited‐state properties according to the time‐dependent density functional theory (TD‐DFT) calculations at PBE0/6–31G(d,p) level. As depicted in Figure S16, the hole and particle distributions of the S_1_ and T_1_ states for **BN‐S** and **BN‐Se** are mostly distributed on the **BCzBN** core, suggesting that these states possess SRCT character. In contrast, the S_2_ and T_2_ of **BN‐S** and **BN‐Se** are more accurately described as having mixed SRCT and LRCT character, and thus distinct orbital types to S_1_/T_1_, providing plausible indirect pathways for RISC.^[^ ^52^, ^53^, ^54^ ^]^ The S_1_/T_1_ states of **BN‐S** and **BN‐Se** are predicted to be 3.18/2.81 and 3.18/2.81 eV by the TD‐DFT calculations, resulting in a large Δ*E*_ST_ of 0.37 eV that don't match well with the experimental measurements (vide infra). Thus, the energies of the excited‐states were modelled at a higher level of theory using the second order algebraic diagrammatic construction Spin‐Component Scaling (ADC2‐SCS) method and the cc‐pVDZ basis set, which has been demonstrated to accurately predict the excited‐state energies of MR‐TADF emitters.^[^ ^38^ ^]^ To reduce the computational cost, we computed the properties of model compounds **MeBN‐S** and **MeBN‐Se** where the peripheral *tert*‐butyl groups have been replaced with methyl groups. Notably, there is essentially no change in the optimized S_0_ structures, electron density distributions, and energies of **MeBN‐S** and **BN‐S**, as well as **MeBN‐Se** and **BN‐Se** (Figures S17 and S18), and so these are appropriate model compounds. This structural change is not expected to lead to significant differences in the optoelectronic properties of monomolecular species in the gas phase calculations. As depicted in Figure 3b, the S_1_/T_1_ energies of **BN‐S** and **BN‐Se** are 2.75/2.61 and 2.74/2.61 eV, leading to a small Δ*E*_ST_s of 0.14 and 0.13 eV, values that are in excellent agreement with experimental results. More importantly, the SOC matrix elements between S_1_ and T_1_, S_1_ and T_2_, S_2_ and T_1_, S_2_ and T_2_ states of **BN‐Se** (0.2501, 0.5380, 2.2723, and 0.7854 cm^−1^) are much higher than those of **BN‐S** (0.0623, 0.1954, 0.1500, and 0.2804 cm^−1^), which are consistent with SOC values based on the X‐ray structures (Figure S11). These results indicate that the introduction of peripheral benzochalcogenophenes at the *para* position to the B of the **tCzBN** moiety is effective in enhancing SOC and thus accelerating RISC.

### Photophysical Properties

**Figure 4:**
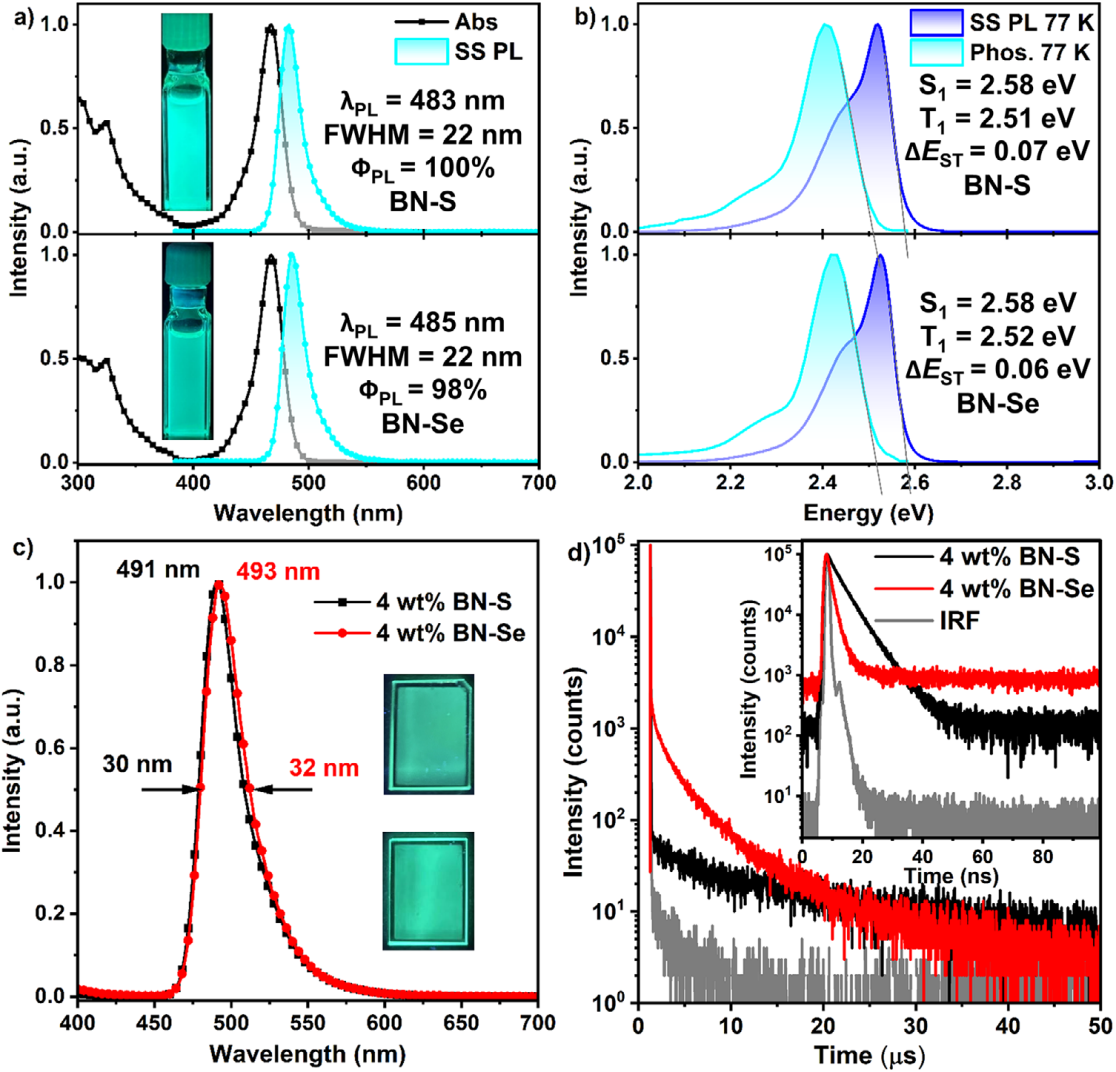
a) Absorption and steady‐state PL (SS‐PL) spectra of **BN‐S** and **BN‐Se** in dilute toluene at room temperature (λ_exc_ = 360 nm); b) Steady‐state PL (77 K) and phosphorescence spectra (1–10 ms, 77 K) of **BN‐S** and **BN‐Se** in toluene (λ_exc_ = 360 nm); c) steady‐state PL spectra and d) Time‐resolved PL decays of **BN‐S** and **BN‐Se** as 4 wt% doped films in PhCzBCz (λ_exc_ = 360 nm).

The ultraviolet‐visible (UV–vis) absorption and steady‐state PL (SS PL) spectra at 77 and 298 K, and phosphorescence spectra at 77 K of **BN‐S** and **BN‐Se** in dilute toluene (10^−5^ M) are shown in Figure 4, and the data collected in Table 1. As depicted in Figure 4a, **BN‐S** and **BN‐Se** possess similar absorption spectra, with intense and sharp absorption bands (molar absorptivities, ε, of 5.2 and 4.6 × 10^4^ M^−1^ cm^−1^) at 468 nm assigned to SRCT transitions, which are close to that of **BCzBN** (Figure S19, *λ*_abs_ of 467 nm in dilute toluene at room temperature, ε of 2.3 × 10^4^ M^−1^ cm^−1^). Upon excitation at 360 nm, the PL spectra of **BN‐S** and **BN‐Se** reflect bright bluish‐green narrowband emission peaking at *λ*_PL_ of 483 and 485 nm, with small FWHM of 22 nm and Stokes shifts of 15 and 17 nm. These compare favorably to those of **BCzBN** (Figure S19, *λ*_PL_ of 482 nm, FWHM: 22 nm, Stokes shift: 15 nm). These suggest that the presence of the chalcogenophene groups does not largely affect the SS PL behavior compared to the parent **BCzBN**. In oxygen‐free toluene solution after bubbling nitrogen for 10 min, **BN‐S** and **BN‐Se** have essentially unity *Φ*_PL_s of 100% and 98%, respectively. Absorption and PL solvatochromic studies are shown in Figure S20. Upon increasing solvent polarity, the absorption spectra of **BN‐S** and **BN‐Se** barely change, indicating that both compounds have a small dipole moment in the ground state. A small bathochromic shift in the emission spectra as a function of solvent polarity was observed, while the relatively high *Φ*_PL_s (> 96%) were conserved and there are mildly larger FWHM values, all of which are consistent with the SRCT emission of **BN‐S** and **BN‐Se** (Table S1). The S_1_ and T_1_ energy levels, calculated from the onsets of the fluorescence and phosphorescence spectra in toluene glass at 77 K (Figure 4b), are 2.58/2.51 eV and 2.58/2.52 eV for **BN‐S** and **BN‐Se**, respectively. This results in moderately small ∆*E*_ST_ of 0.07 and 0.06 eV, respectively, comparable to 0.14 eV for **BCzBN**.^[^ ^51^ ^]^ The transient decay behavior in oxygen‐free toluene solution (10^−5^ M) was also examined (Figure S21 and Table S2). **BN‐S** exhibits biexponential decay kinetics, with a prompt fluorescence (*τ*_PF_) lifetime of 6.8 ns and a delayed fluorescence (*τ*_DF_) lifetime of 2.7 µs. **BN‐Se** has shorter *τ*_PF_ of 2.7 ns and *τ*_DF_ of 0.7 µs. Combined with the high *Φ*_PL_ values of 100% and 98% in oxygen‐free toluene solution, the calculated *k*_RISC_ of **BN‐S** and **BN‐Se** are very fast at 1.2 × 10^6^ s^−1^ and 7.6 × 10^6^ s^−1^ using the methodology of Tsuchiya et al.^[^ ^55^ ^]^ The fast *k*_RISC_ is beneficial and should directly translate into devices showing suppressed efficiency roll‐off.

**Table 1:** Summary of the Photophysical Properties of BCzBN, BN‐S, and BN‐Se.

	λ_abs_ ^a)^	λ_PL_ ^b)^	FWHM^c)^	Stokes shift^d)^	S_1_ ^e)^	T_1_ ^f)^	Δ*E*_ST_ ^g)^	Φ_PL_ ^h)^	τ_PF_ ^i)^	τ_DF_ ^j)^	*k*_RISC_ ^k)^
Emitters	(nm)	(nm)	(nm)	(nm)	(eV)	(eV)	(eV)	(%)	(ns)	(µs)	(10^5^ s^−1^)
**BCzBN**	467	482/490	22/29	15	2.65	2.53	0.12	94/92	5.0	122	0.14
**BN‐S**	468	483/493	22/34	15	2.58	2.51	0.07	100/99	5.2	11.5	2.5
**BN‐Se**	468	485/493	22/32	17	2.58	2.52	0.06	98/96	2.2	3.5	7.2

^a)^ Absorption maximum of lowest energy band in diluted toluene.

^b)^ Emission maximum in toluene and 4 wt% doped films in PhCzBCz.

^c)^ FWHM in toluene and 4 wt% doped films in PhCzBCz.

^d)^ Stokes shift in toluene.

^e)^ The energy of S_1_ measured in toluene.

^f)^ The energy of T_1_ measured in toluene.

^g)^ The Δ*E*_ST_ measured in toluene.

^h)^ Absolute *Φ*_PL_ in toluene and 4 wt% doped films in PhCzBCz measured using an integrating sphere.

^i)^ The prompt fluorescence lifetimes.

^j)^ The delayed fluorescence lifetimes.

^k)^ The rate constant for RISC.

To verify the role of the pendant benzochalcogenophene in alleviating the aggregation‐caused quenching (ACQ), the PL spectra of the emitters in a suitably high triplet energy host, PhCzBCz, at concentrations ranging from 1 to 12 wt% were studied (Figure S22). The PL spectra of **BN‐S** and **BN‐Se** did not change significantly, still retaining small FWHM values of < 35 nm and high *Φ*_PL_ values of >90%. With an eye to their use as emitters in OLEDs, we next turned our attention to the characterization of these two compounds in films at 4 wt% doping owing to the highest *Φ*_PL_ value recorded at this doping level. The PL spectra of both compounds are slightly broader and red‐shifted compared with the emission spectra in toluene solutions due to intermolecular interactions in the film (Figure 4c). The high *Φ*_PL_ is conserved, with **BN‐S** and **BN‐Se** having values of 99% and 96%, respectively. As shown in Figure 4d, the time‐resolved PL measurements of **BN‐S** and **BN‐Se** show biexponential decay profiles, with the *τ*_PF_s of 5.2 and 2.2 ns and *τ*_DF_s of 11.5 and 3.5 µs, respectively. Compared to the parent molecule **BCzBN** (Figure S19, *τ*_DF_: 122 µs, in 4 wt% doped film in PhCzBCz) and **BN‐S**, **BN‐Se** exhibits a significantly shorter *τ*_DF_, reflecting a faster RISC process in this compound. Temperature‐dependent time‐resolved PL decay measurements for **BN‐S** and **BN‐Se** reveal the expected increase in the delayed emission with increasing temperature (Figure S23), confirming that these two compounds are TADF. The key kinetics parameters were calculated using the similar methodology (Table S3).^[^ ^55^ ^]^ Reflecting their near unity *Φ*_PL_s, the radiative rate constants (*k*_r_) of **BN‐S** and **BN‐Se** are high at 0.7 × 10^8^ and 1.7 × 10^8^ s^−1^, respectively. The *k*_RISC_ of **BN‐Se** (7.2 × 10^5^ s^−1^) is ∼2.9 times faster than that of **BN‐S** (2.5 × 10^5^ s^−1^), ∼51.4 times faster than **BCzBN** (1.4 × 10^4^ s^−1^) and 12 times faster than **BNDBT** (6.0 × 10^4^ s^−1^, a dibenzothiophene‐incorporated MR‐TADF emitter from our previous work^[^ ^43^ ^]^), this mainly being due to the heavy atom effect driving larger SOC. In addition, angle‐dependent PL measurements reveal that the transition dipole moments of the 4 wt% films of **BN‐S** and **BN‐Se** are strongly horizontally aligned, with emitting dipole ratios (Θ) of 96.5% and 92.5%, respectively (Figure S24). Thus, it is expected that the light out‐coupling efficiency in the device would be high. The calculated S_1_→S_0_ transition dipole moments (TDMs) of **BN‐S** and **BN‐Se** are oriented mainly along the x‐y plane and the TDM vectors have minor z components (Figure S25), suggesting that both emitters have a natural tendency to preferentially align their molecular axis horizontally during the deposition process, which is conducive to enhancing the optical out‐coupling efficiency and boosting OLED performance.

### Electroluminescence Properties

**Figure 5:**
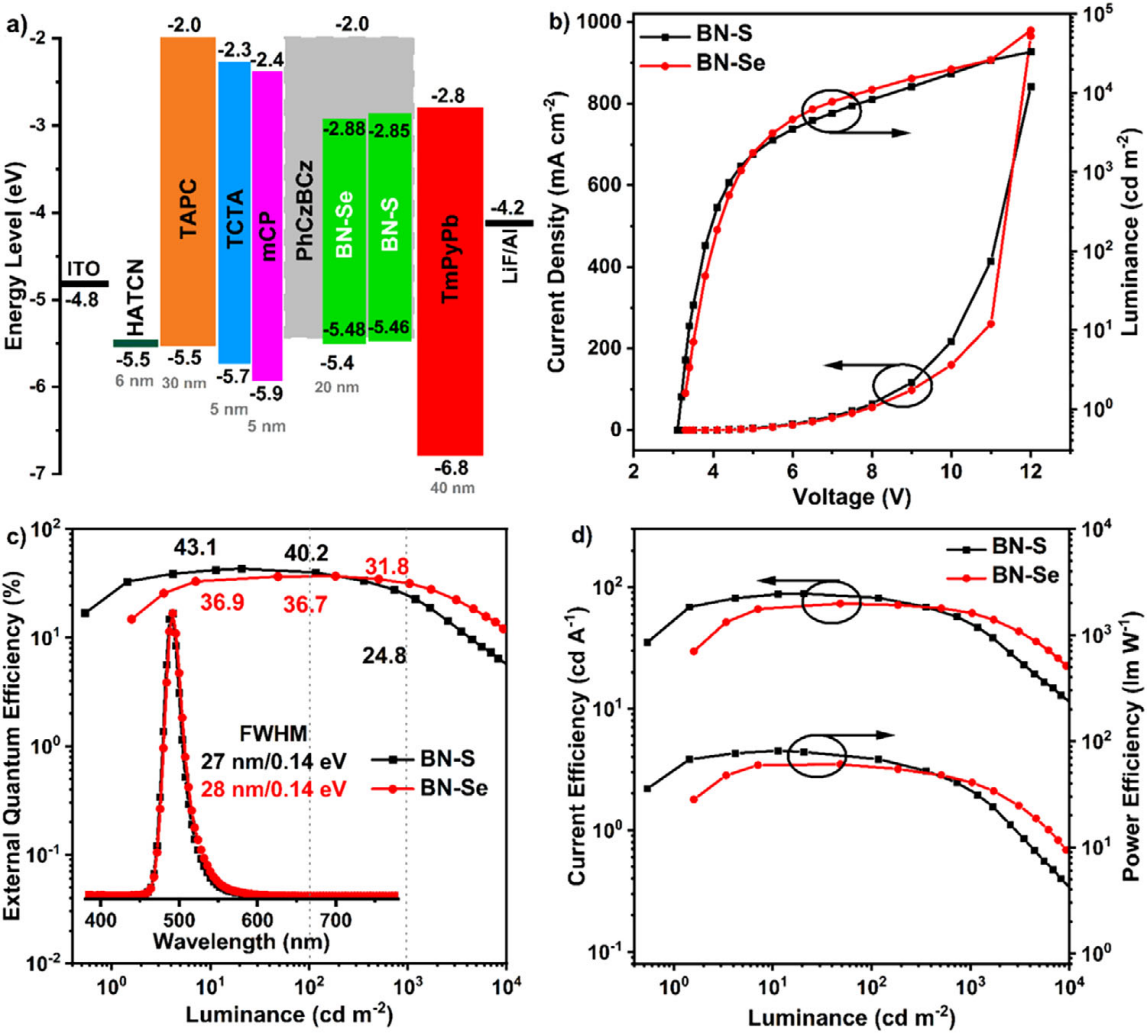
a) The energy level diagram in the devices; b) luminance‐voltage‐current density curves, c) EQE versus luminance curves (inset: the EL spectra), and d) CE/PE versus luminance curves characteristics of the **BN‐S** and **BN‐Se**‐based devices at 4 wt% doping concentration.

Vacuum‐deposited OLEDs were fabricated with the stack of ITO/HATCN (5 nm)/TAPC (30 nm)/TCTA (10 nm)/PhCzBCz: *x* wt% of **BN‐S** and **BN‐Se** (20 nm)/TPBi (40 nm)/LiF (1 nm)/Al (120 nm) (*x *= 1, 4, 8, and 12), Figures 5a and S26. Because of the high triplet energy, ambipolar charge transport ability, and high *Φ*_PL_ of the emitters in PhCzBCz, this host was selected for these devices.^[^ ^50^ ^]^ Four doping concentrations (1, 4, 8, and 12 wt%) were assessed for the **BN‐S** and **BN‐Se** to identify a concentration where there is effective energy transfer from the host to the emitter. All devices displayed relatively low turn‐on voltages (V_on_) of 3.1–3.4 V and extremely high maximum luminance (L_max_) exceeding 30,000 cd m^−2^, pointing to effective charge injection and transport in the devices. In contrast to the **BCzBN**‐based device,^[^ ^51^ ^]^ no obvious bathochromic shifting nor spectral broadening were observed in the OLEDs using the **BN‐S** and **BN‐Se** as emitters at doping concentrations of up to 12 wt% (Figure S27), implying that these emitters adopt geometries that minimize intermolecular interactions.

**Figure 6:**
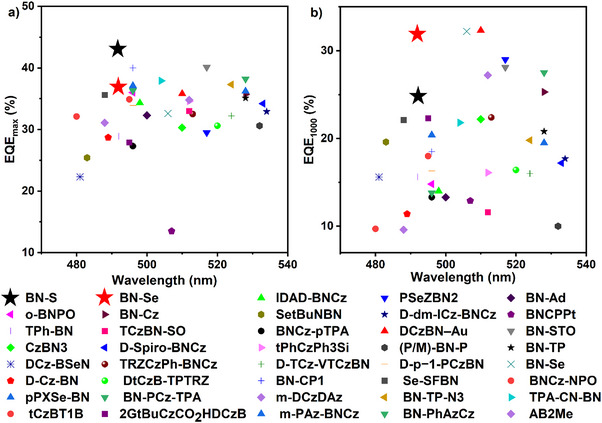
a) EQE_max_ versus EL wavelength of mono‐boron MR‐TADF OLEDs with emission peak between 470–540 nm; b) EQE@1000 cd m^−2^ versus EL wavelength of mono‐boron MR‐TADF OLEDs with emission peak between 470–540 nm. See Table S2 for details.

The device performances including luminance‐voltage‐current density, EL spectra and EQE/CE/PE versus luminance curves characteristics at different dopant concentrations are depicted in Figures S27–S29, and the detailed device parameters are compiled in Table 2. Both devices achieved the best EL performances when the doping ratio was 4 wt%, owing to the highest *Φ*_PL_ values. As shown in Figure 5b–d, the devices with **BN‐S** and **BN‐Se** exhibited blue‐green emission peaking at *λ*_EL_ of 492 nm, with associated Commission International de l’Éclairage, CIE, coordinates of (0.09, 0.42) and (0.09, 0.43), and narrow FWHM of 27 and 28 nm. Moreover, the EL spectrum was stable to changes in applied bias voltage (Figures S30–S32), demonstrating that the EL emission originates uniquely from monomer emission of the emitters in the EMLs and there is no excimer or exciplex emission. Without using any light out‐coupling enhancement or additional sensitizer, the **BN‐S**‐based device exhibited the best EL performances with an ultrahigh brightness of 33,123 cd m^−2^, a remarkable EQE_max_ of 43.1%, a maximum current efficiency (CE_max_) of 88.3 cd A^−1^, and a maximum power efficiency (PE_max_) of 88.1 lm W^−1^. These impressive device metrics are associated with the synergy of efficient exciton harvesting, high *Φ*_PL_, and strong light outcoupling efficiency. To the best of our knowledge, this EQE_max_ is the highest reported for mono‐boron MR‐TADF devices without sensitization (i.e., so‐called hyperfluorescence or hyperphosphorescence) devices (Figure 6a and Table S4). However, despite the reasonably fast *k*_RISC_, the devices exhibited serious efficiency roll‐off at higher luminance, with EQEs of 40.2% at 100 cd m^−2^ and 24.8% at 1000 cd m^−2^. Notably, the **BN‐Se**‐based device also exhibited outstanding device efficiencies, with an EQE_max_ of 36.9%, CE_max_ of 73.4 cd A^−1^, PE_max_ of 60.7 lm W^−1^, and L_max_ of 61814 cd m^−2^. Owing to the faster *k*_RISC_, the devices impressively exhibited negligible efficiency roll‐off, with EQEs of 36.7% at 100 cd m^−2^ and 31.8% at 1000 cd m^−2^ (Figure 6b and Table S4).

**Table 2:** Summary of the EL data for the **BN‐S** and **BN‐Se**‐based devices at different dopant concentrations.

Emitters	λ_EL_ ^a^ ^)^ (nm)	FWHM^b^ ^)^ (nm)	V_on_ ^c^ ^)^ (V)	L_max_ (cd m^−2^)	CE^d^ ^)^ (cd A^−1^)	PE^e^ ^)^ (lm W^−1^)	EQE^f^ ^)^ (%)	Roll‐off^g^ ^)^ (%)	CIE^h^ ^)^ (x, y)
BN‐S (1 wt%)	492	27	3.2	42 366	85.0/77.2/45.3	76.3/61.8/29.2	40.7/37.9/22.2	6.9/45.4	(0.09, 0.40)
BN‐S (4 wt%)	492	27	3.1	33 123	88.3/82.3/50.5	81.1/68.6/34.7	43.1/40.2/24.8	6.7/42.7	(0.09, 0.42)
BN‐S (8 wt%)	492	28	3.2	35 266	79.9/73.4/43.6	71.7/60.5/29.4	38.3/35.2/21.3	8.1/44.4	(0.10, 0.46)
BN‐S (12 wt%)	492	30	3.2	28 655	90.2/86.4/47.1	79.2/69.8/31.3	36.7/35.4/19.7	3.5/46.3	(0.11, 0.49)
BN‐Se (1 wt%)	492	27	3.4	39 547	66.5/63.1/57.3	51.0/49.1/38.0	36.6/34.7/31.6	5.2/13.6	(0.09, 0.42)
BN‐Se (4 wt%)	492	28	3.3	61 814	73.4/72.4/61.6	60.7/57.5/41.3	36.9/36.7/31.8	0.5/13.8	(0.09, 0.43)
BN‐Se (8 wt%)	492	28	3.4	44 976	69.2/69.0/54.9	56.5/52.8/35.7	36.8/36.7/29.7	0.3/19.3	(0.09, 0.44)
BN‐Se (12 wt%)	492	29	3.4	51 699	70.1/69.5/57.4	57.9/53.6/37.8	36.4/36.1/30.8	0.8/15.4	(0.10, 0.46)

^a)^ Electroluminescence peak at 5 V.

^b)^ The FWHM of EL spectra.

^c)^ Turn‐on voltage at 1 cd m^−2^.

^d)^ CE of maximum/at 100 cd m^−2^/1000 cd m^−2^.

^e)^ PE of maximum/at 100 cd m^−2^/1000 cd m^−2^.

^f)^ EQE of maximum/at 100 cd m^−2^/1000 cd m^−2^.

^g)^ EQE roll‐off at 100 cd m^−2^/1000 cd m^−2^.

^h)^ CIE coordinates at 5 V.

We investigated the angle‐dependent EL intensities of the devices with **BN‐S** and **BN‐Se**. The light distribution of both devices is nearly Lambertian with corresponding Lambertian coefficients of 0.96 and 0.98 for the devices with **BN‐S** and **BN‐Se**, respectively (Figure S33), confirming that the high EQE values are not overestimated. To confirm the reproducibility and reliability of the device performance, eight groups of devices with each of the two emitters were fabricated in parallel (4 wt% doping concentration). The EQE–luminance curves of the two family of devices were essentially consistent (Figure S34), demonstrating that the results are reproducible. Operational lifetimes of the devices with **BN‐S** and **BN‐Se** under an initial luminance of 1000 cd m^−2^ were measured (Figure S35). The LT_50_ values (lifetime to 50% of the initial luminance) were 10.0 and 5.23 h for the devices with **BN‐S** and **BN‐Se**, respectively, which places them as some of the most stable sulfur and selenium‐containing MR‐TADF OLEDs reported to date (Figure S36 and Table S5).^[^ ^34^, ^35^, ^36^, ^37^, ^39^, ^56^ ^]^

## Conclusions

Here we demonstrate that decorating a benzochalcogenophene *para* to the boron atom of a known MR‐TADF core, **tCzBN**, can significantly improve the photophysical properties, particularly accelerating *k*_RISC_ and mitigating ACQ, without affecting the emission energy. Based on this study, it can be concluded that our molecular design provides an effective approach to devices showing very high EQEs and that overcome significant efficiency roll‐off in MR‐TADF OLEDs. This is because the emitters in the host show simultaenously high *Φ*_PL_, fast *k*_RISC_, and strongly horizontal orientation of their TDMs. Our results clearly demonstrate the value of these benzochalcogenophene substituents in significantly improving device performance without adversely affecting the color point.

## Supporting Information

^1^H NMR and ^13^C NMR spectra, MS of all target compounds; supplementary computational data and coordinates; Crystallographic data (CIF). Additional photophysical and OLED data.

## Author Contributions

P. Lu conceived the project and experiments. F.L. and Z.S. conducted the experiment and wrote the manuscript. Z.C. fabricated the OLEDs. D.C., Y.X., and W.D. conducted the theoretical calculations, Y.Y. and L.W. conducted the photophysical characterizations and performed the data analysis. E.Z.‐C. analyzed the data and revised the manuscript. All the authors contributed to writing the manuscript.

## Conflict of Interests

The authors declare no conflict of interest.

## Supporting information



Supporting Information

Supporting Information

Supporting Information

## Data Availability

The research data supporting this publication can be accessed at https://doi.org/10.17630/eb7052d3‐8774‐4814‐83fb‐569359a53961.
