# Supplementary material for: Judicious Use of Chalcogens in Multiresonant Thermally Activated Delayed Fluorescent Emitters Leads to OLEDs with Efficiencies Exceeding 36% and Showing Mild Efficiency Roll‐Off
Source: Angew Chem Int Ed Engl. 2025 Aug 6;64(39):e202511866. doi: 10.1002/anie.202511866 (PMC12455388; doi:10.1002/anie.202511866)
Supplement: Supplementary file 1 — Supporting Information [file ANIE-64-e202511866-s002.pdf]

Supporting Information

Judicious Use of Chalcogens in Multiresonant Thermally Activated Delayed Fluorescent Emitters Leads to OLEDs with Efficiencies Exceeding 36% and Showing Mild Efficiency Roll-Off

Futong Liu[a], Zihan Su[a], Zhuang Cheng[a], Dongyang Chen[b], Yangze Xu[a], Yan Yan[a], Wei Dong[a],

Liang Wan[a], Eli Zysman-Colman[b] and Ping Lu*[a]*

[a] State Key Laboratory of Supramolecular Structure and Materials, Department of Chemistry, Jilin University, Changchun 130012 (P. R. China). E-mail: lup@jlu.edu.cn.

[b] Organic Semiconductor Centre, EaStCHEM School of Chemistry, University of St Andrews, St Andrews, UK, KY16 9ST. E-mail: eli.zysman-colman@st-andrews.ac.uk.

Contents

1. Experimental section S2

1.1 General information S2

1.2 Electrochemical measurements S2

1.3 Device fabrications and measurements S3

1.4 Theoretical calculations S4

1.5 Calculation Formulas for the Photophysical Parameters S5

1.6 Synthetic procedures S6

2. Supplementary Figures and Tables S8

3. References S28

1. Experimental section

1.1 General information

All the reagents and solvents were used as received without further purification. ^{1}H NMR, ^{11}B NMR, and ^{13}C NMR were recorded by Bruker AVANCE 500 spectrometer at 500 MHz or 125 MHz with tetramethylsilane (TMS) as the internal standard. Elemental analysis was carried out on a Flash EA 1112, CHNSO elemental analysis instrument. MALDI-TOF-MS mass spectra were obtained from an AXIMA-CFRTM plus instrument. Thermal gravimetric analysis (TGA) was performed on a Perkin-Elmer thermal analysis system from 30 °C to 900 °C. Differential scanning calorimetry (DSC) was measured by NETZSCH (DSC-204) unit from 30 °C to 400 °C. Organic films for optical measurements were fabricated by thermal evaporation under high vacuum onto clean quartz substrates. UV-Vis absorption spectra were recorded on a Shimadzu UV-3100 spectrophotometer. Steady-state photoluminescence (PL) spectra were measured on an RF-5301PC spectrophotometer. Time-resolved photoluminescence measurements were performed on Edinburgh spectrometer LP980 365-nm laser flash as excitation source. The PL lifetime was measured using an FLS980 spectrometer with a 375-nm picosecond pulsed light-emitting diode excitation source (pulse width: 898.3 ps). Edinburgh FLS980 steady state fluorimeter equipping with an integrating sphere was employed to measure the absolute photoluminescence quantum yields (Φ_{PL}s) of both solution and films at room temperature.

1.2 Electrochemical measurements

Cyclic voltammetry (CV) was measured using a BAS 100B/W electrochemical analyzer with a standard one-compartment, three-electrode electrochemical cell. Tetrabutylammoniumhexafluorophosphate ($TBAPF_6$) in anhydrous dimethyl formamide (DMF) or anhydrous dichloromethane (0.1 M) was used as the electrolyte

for negative or positive scans. The working electrode was a glass-carbon disk electrode. The counter electrode was a Pt wire. The reference electrode was Ag/Ag^+. A ferrocenium/ferrocene (Fc^+/Fc) redox couple was used as the internal standard, and the formal potential of Fc^+/Fc is 4.8 eV below vacuum. All potentials relative to Ag/Ag^+ electrode obtained from CV measurement were referenced against Fc^+/Fc to calculate HOMO/LUMO levels. As a result, the Ag/Ag^+ electrode is just a pseudo-reference. The HOMO/LUMO levels are calculated according to the following formalism:

$$\text{HOMO} = -(E_{\text{ox}} \text{ vs. } Fc^+/Fc + 4.8) \text{ eV} \quad (S1)$$

$$\text{LUMO} = -(E_{\text{red}} \text{ vs. } Fc^+/Fc + 4.8) \text{ eV} \quad (S2)$$

where the E_{ox} vs. Fc^+/Fc and E_{red} vs. Fc^+/Fc are oxidation and reduction onset potentials relative to Fc^+/Fc reference, respectively.

1.3 Device fabrications and measurements

The bottom emission device structure of the MR-TADF OLED used was indium tin oxide (ITO)/HATCN (5 nm)/TAPC (30 nm)/TCTA (10 nm)/x wt% emitters in PhCzBCz (30 nm)/TPBi (40 nm)/LiF (1 nm)/Al (120 nm), where ITO, HATCN (1,4,5,8,9,11-hexaazatriphenylene-hexacarbonitrile), TAPC (di-[4-(*N*,*N*-ditolyl-amino)-phenyl] cyclohexane), TCTA (4,4',4''-tri(*N*-carbazolyl)-triphenylamine), TPBi (1,3,5-tris(1-phenyl-1*H*-benzimidazol-2-yl)benzene), LiF (lithium fluoride) and Al were employed as the anode, hole injection layer, hole transport layer, exciton-blocking layer, electron transport layer, electron injection layer and cathode, respectively. PhCzBCz (9-(2-(9-phenyl-9*H*-carbazol-3-yl)phenyl)-9*H*-3,9'-bicarbazole) was chosen as the host for the MR-TADF emitters. ITO coated glasses with a sheet resistance of 20 Ω $square^{-1}$ were used as the substrate and cleaned by deionized water, isopropyl alcohol, acetone and toluene. Then the ITO glasses were irradiated in UV-zone for 30 min. The deposition system for organic and metal deposition has a base pressure lower than 5×10^{-6} mbar. The hole injecting layer HATCN was deposited at 0.1 Å s^{-1}. The deposition rate of all other organic layers was 1.0 Å s^{-1}. The electron injecting layer LiF was deposited at a rate of 0.1 Å s^{-1} and then the capping Al metal layer was deposited at a rate of 4.0 Å s-

[1]. The electroluminescent (EL) characteristics were measured using a Keithley 2400 programmable electrometer and a PR-650 Spectroscan spectrometer under ambient condition at room temperature. The emitting dipole orientation ratio (Θ//) of the emitting films was determined by least-square fitting of the measured angle-dependent p-polarized emission intensity to the calculated results. The device operational lifetimes (LT_{50}) were evaluated by measuring the luminance over time at a constant current densities starting from an initial luminance of 1000 cd m^{-2}.

1.4 Theoretical calculations

The calculations were performed using either Density Functional Theory (DFT) implemented within Gaussian 16, or the second order algebraic diagrammatic construction Spin-Component Scaling (ADC2-SCS) method[1] using the Turbomole/7.5 package.[2] We optimized the ground state using the PBE0[3] functional with the 6-31G(d,p)[4] basis set. The excited-state calculations were performed at Time-dependent DFT (TD-DFT)[5] using the same functional and basis set as for ground-state geometry optimization. For the ADC(2) calculations, the excited-states were computed using ADC2-SCS with the ccpVDZ basis set,[6] computing the two first singlet (S_1 and S_2) and two first triplet excited states (T_1 and T_2)[7]. All calculations were submitted and processed using in-house developed software Digichem ver 6.[8] PySOC[9] was used for the calculation of the spin-orbit coupling matrix element (SOCME) values based on the optimized T_1 geometries. The noncovalent interactions were analyzed through reduced density gradient (RDG) analysis based on the optimized S_1 geometry using Multiwfn 3.6[10] and visualized with the visual molecular dynamics (VMD) program[11]. The bond dissociation energy (BDE) was calculated according to the enthalpy change for the corresponding reaction of the homolytic cleavage of a single bond in the gas phase at 298 K and 1 atm.

1.5 Calculation Formulas for the Photophysical Parameters

Fitting of the time-resolved luminescence measurements

Time-resolved PL measurements were fitted to a sum of exponentials decay model, with chi-squared (χ^2) values between 1 and 2, using the FLS980 spectrometer. Each component of the decay is assigned with a weight, (wi), which is the contribution of the emission from each component to the total emission.

The average lifetime was then calculated using the following expressions:

1. Two exponential decay model:

$$\tau_{AVG} = \tau_1 w_1 + \tau_2 w_2 \quad \text{(S3)}$$

with weights defined as $w_1 = \frac{A_1\tau_1}{A_1\tau_1 + A_2\tau_2}$ and $w_2 = \frac{A_2\tau_2}{A_1\tau_1 + A_2\tau_2}$ where A_1 and A_2 are the preexponential-factors of each component.

2. Three exponential decay model:

$$\tau_{AVG} = \tau_1 w_1 + \tau_2 w_2 + \tau_3 w_3 \quad \text{(S4)}$$

with weights defined as $w_1 = \frac{A_1\tau_1}{A_1\tau_1 + A_2\tau_2 + A_3\tau_3}$, $w_2 = \frac{A_2\tau_2}{A_1\tau_1 + A_2\tau_2 + A_3\tau_3}$ and $w_3 = \frac{A_3\tau_3}{A_1\tau_1 + A_2\tau_2 + A_3\tau_3}$ where A_1, A_2 and A_3 are the preexponential-factors of each component.

The rate constants for radiative decay (k_r) and nonradiative decay (k_{nr}) from S_1 to S_0 states, the rate constants of intersystem crossing (k_{ISC}) and reverse intersystem crossing (k_{RISC}) were calculated from the following six equations:

$$k_p = 1/\tau_p \quad \text{(S5)}$$

$$k_d = 1/\tau_d \quad \text{(S6)}$$

$$k_r = \Phi_p k_p + \Phi_d k_d \approx \Phi_p k_p \quad \text{(S7)}$$

$$k_{nr} = (1 - \Phi_{PL})\, k_r/\Phi_{PL} \quad \text{(S8)}$$

$$k_{ISC} = k_p - k_r - k_{nr} \quad \text{(S9)}$$

$$k_{RISC} = k_p\, k_d\, \Phi_d/(k_{ISC}\, \Phi_p) \quad \text{(S10)}$$

Where τ_p and τ_d represent the prompt and decay fluorescence lifetime, which determined from transient PL spectra. The k_p and k_d represent the decay rate constants

for prompt and delayed fluorescence, respectively. Φ_p and Φ_d indicate prompt and delayed fluorescence components and can be distinguished from the total Φ_{PL} by comparing the integrated intensities of prompt and delayed components in the transient PL spectra.

1.6 Synthetic procedures

All the reagents and solvents used for the syntheses were purchased from Aldrich and Acros and used as received. The precursors **SeBr** and **DtCzB-Bpin** were prepared in high yield according to the reported literature procedures.[12,37]

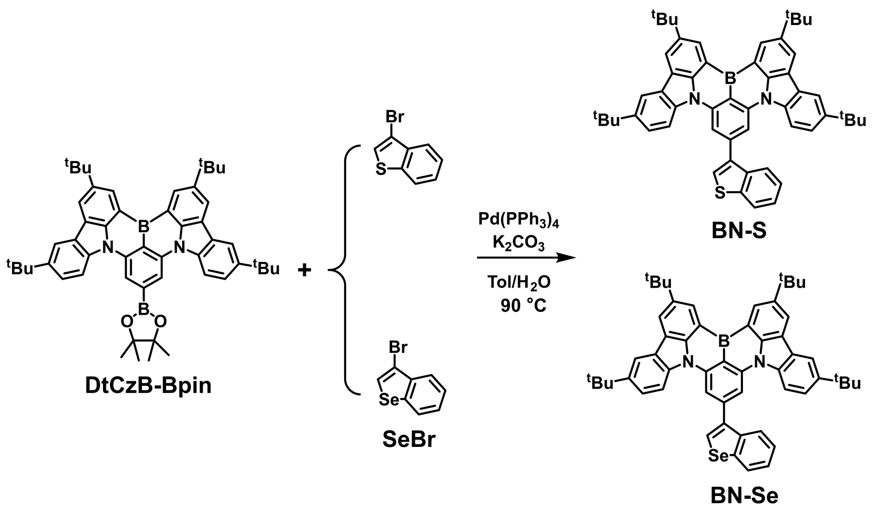

Scheme S1. Synthetic procedures of **BN-S** and **BN-Se**.

Synthesis of BN-S: In a 100 mL round flask, a mixture of **DtCzB-Bpin** (766 mg, 1 mmol), 3-bromothianaphthene (256 mg, 1.2 mmol), K_2CO_3 (5.52 g, 40 mmol), $Pd(PPh_3)_4$ (230 mg, 0.2 mmol), 20 mL distilled water and 40 mL toluene was heated to reflux and stirred under N_2 for 48 hours. The mixture was washed thrice with 30 mL water and extracted with dichloromethane. The organic phase was collected and dried with anhydrous Na_2SO_4, and the solvent was evaporated. The residue was purified via column chromatography eluting with petroleum ether-dichloromethane (1:1, v/v) mixtures to afford a yellow solid. (520 mg, **Yield**: 67%). **^{1}H NMR (500 MHz,**

$CDCl_3$) δ (ppm): 9.19 (s, 2H), 8.58 (s, 2H), 8.51 (s, 2H), 8.40 (d, *J* = 8.8 Hz, 2H), 8.37 – 8.28 (m, 3H), 8.09 (d, *J* = 8.0 Hz, 1H), 7.81 (s, 1H), 7.64 (d, *J* = 8.8 Hz, 2H), 7.56 (m, 3H), 1.71 (s, 18H), 1.54 (s, 18H); **^{13}C NMR (126 MHz, $CDCl_3$) δ (ppm):** 145.43, 144.70, 141.74, 141.01, 140.67, 138.78, 138.33, 138.08, 129.86, 127.16, 124.89, 124.54, 123.69, 123.25, 123.08, 121.71, 120.72, 117.32, 114.15, 108.40, 35.22, 34.81, 32.22, 31.83; **HRMS (MALDI-TOF):** $C_{54}H_{53}BN_2S$ **Calculated:** 772.4; **Found:** 771.9. **Anal. Calcd** (%) for $C_{54}H_{53}BN_2S$: C, 83.92; H, 6.91; N, 3.62; **Found:** C, 83.90; H, 6.92; N, 3.60.

Synthesis of BN-Se: **BN-Se** was synthesized according to the same procedure as for **BN-S** by using Se-Br (307 mg, 1.2 mmol) instead of 3-bromothianaphthene. **BN-Se** (601 mg, **Yield:** 73%) was obtained as a yellow solid. **^{1}H NMR (500 MHz, $CDCl_3$) δ (ppm):** 9.19 (s, 2H), 8.53 (t, *J* = 8.0 Hz, 4H), 8.37 (d, *J* = 8.7 Hz, 2H), 8.28 (dd, *J* = 14.1, 7.0 Hz, 4H), 8.14 (d, *J* = 7.9 Hz, 1H), 7.62 (d, *J* = 8.7 Hz, 2H), 7.55 (t, *J* = 7.5 Hz, 1H), 7.52 – 7.46 (m, 1H), 1.72 (s, 18H), 1.54 (s, 18H); **^{13}C NMR (126 MHz, $CDCl_3$) δ (ppm):** 145.42, 144.76, 144.53, 142.27, 141.74, 140.58, 138.32, 129.86, 127.52, 127.13, 126.36, 125.78, 124.85, 124.54, 123.70, 122.45, 121.72, 120.73, 117.29, 114.18, 108.61, 35.22, 34.81, 32.23, 31.83. **HRMS (MALDI-TOF):** $C_{54}H_{53}BN_2Se$ **Calculated:** 820.3; **Found:** 819.8. **Anal. Calcd** (%) for $C_{54}H_{53}BN_2Se$: C, 79.12; H, 6.52; N, 3.42; **Found:** C, 79.15; H, 6.53; N, 3.41.

2. Supplementary Figures and Tables

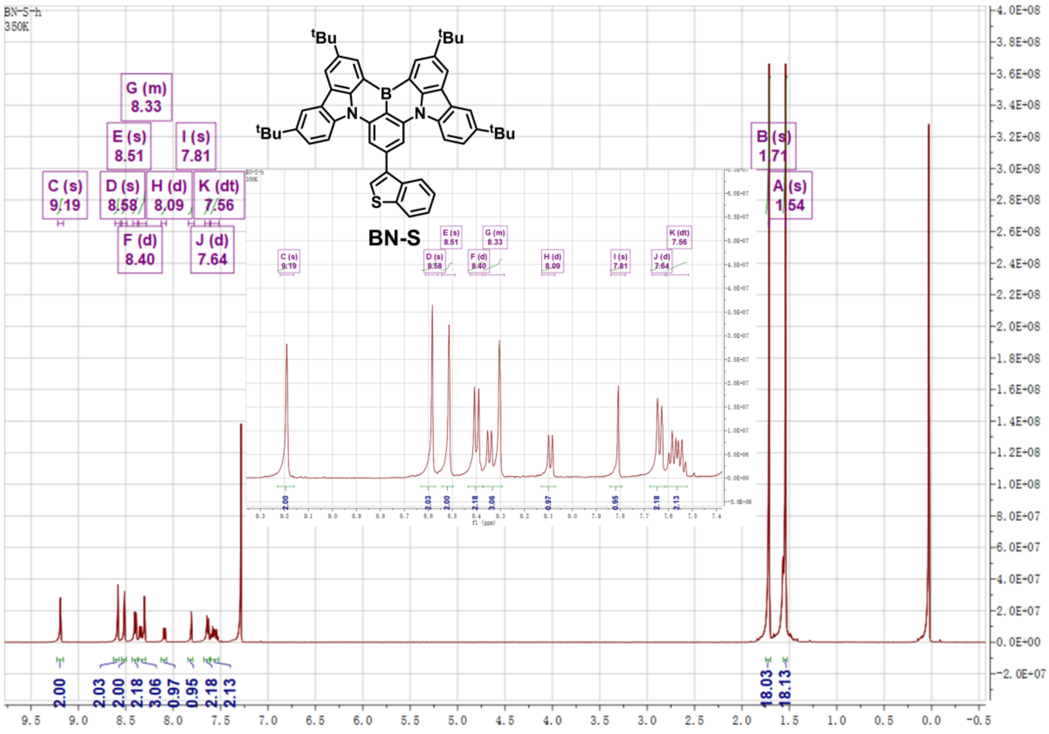

Figure S1. ^{1}H NMR spectrum of **BN-S** measured in $CDCl_3$.

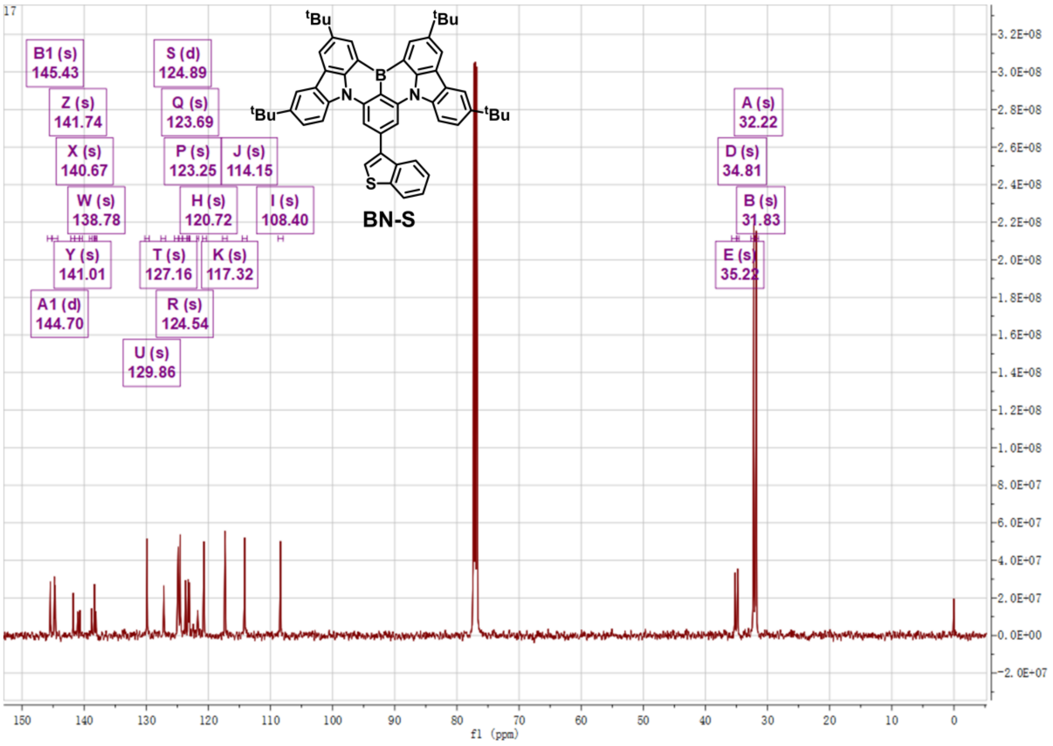

Figure S2. ^{13}C NMR spectrum of **BN-S** measured in $CDCl_3$.

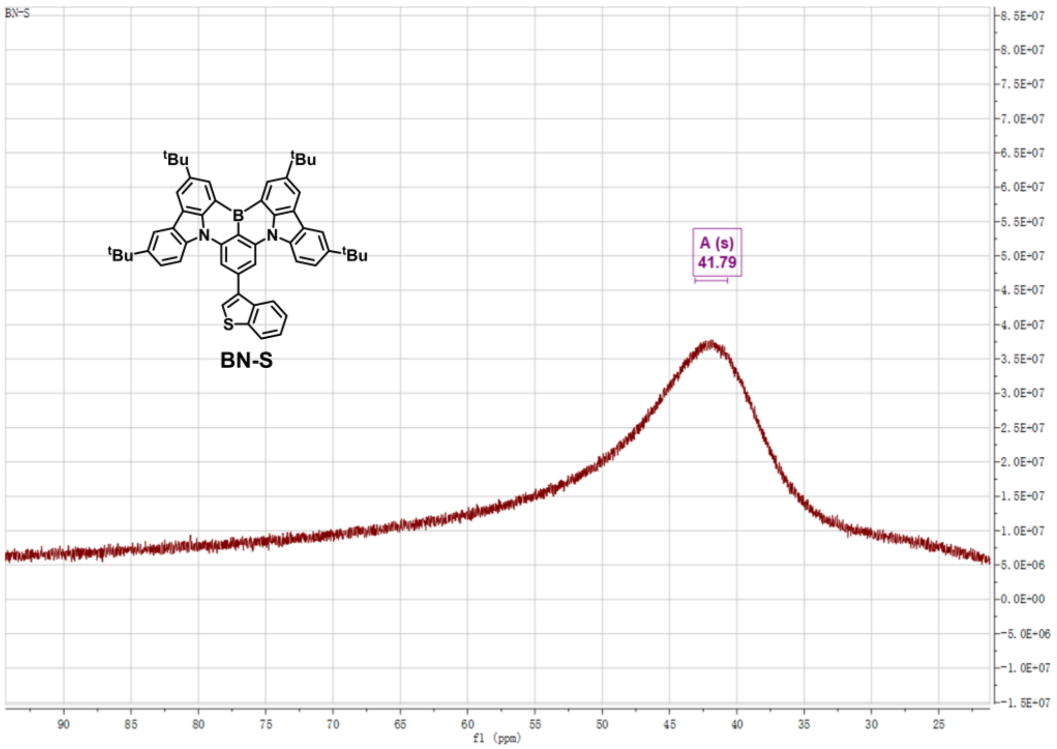

Figure S3. ^{11}B NMR spectrum of **BN-S** measured in CD_2Cl_2.

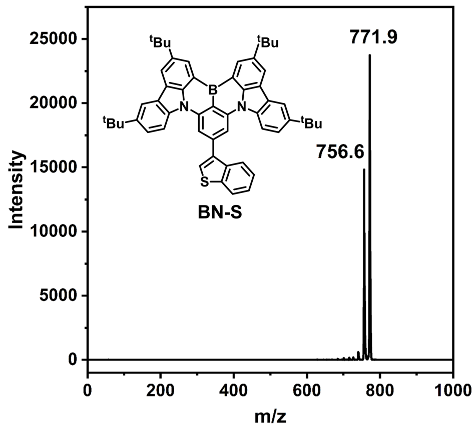

Figure S4. MALDI-TOF MS of **BN-S.**

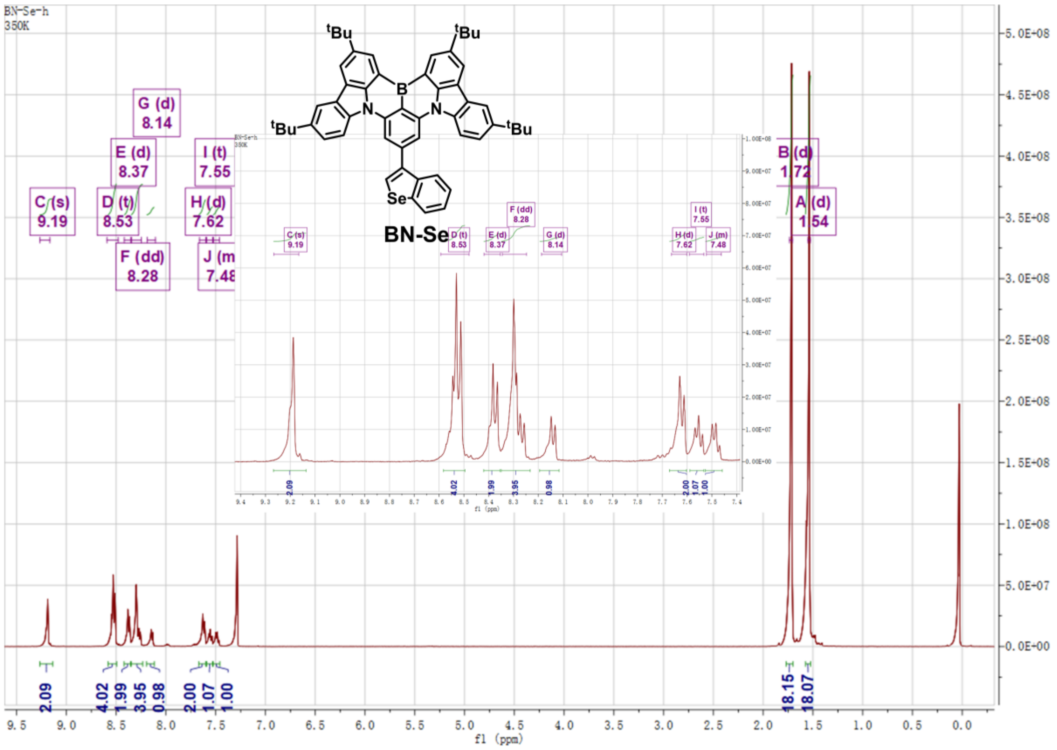

Figure S5. ^{1}H NMR spectrum of **BN-Se** measured in $CDCl_3$.

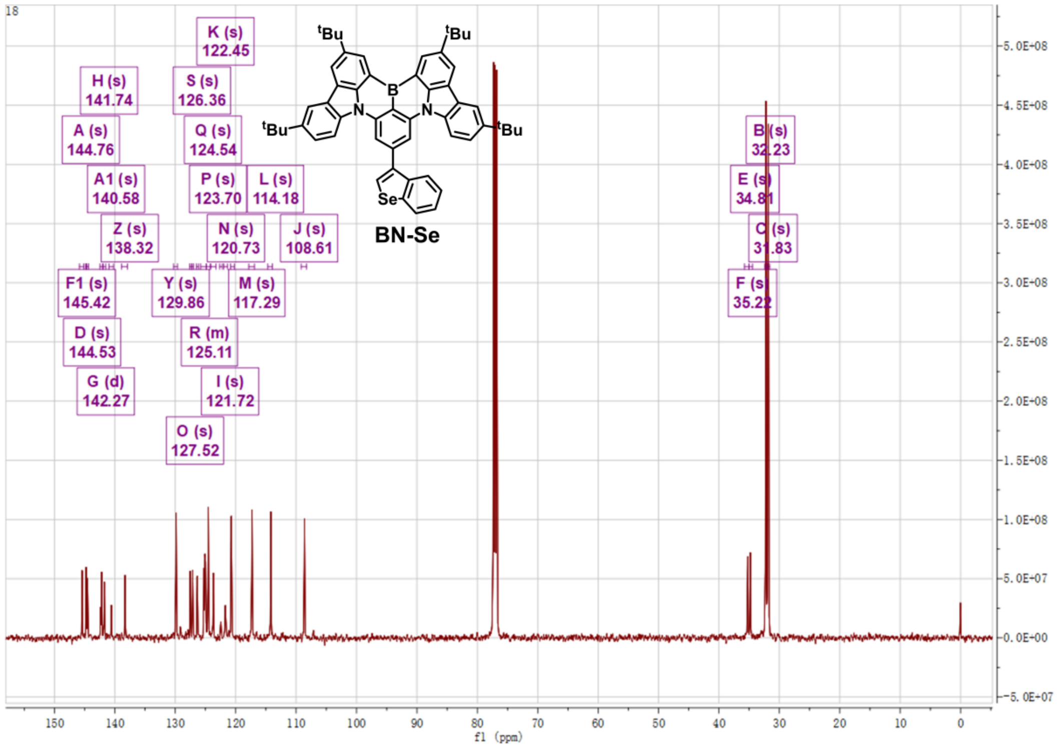

Figure S6. ^{13}C NMR spectrum of **BN-Se** measured in $CDCl_3$.

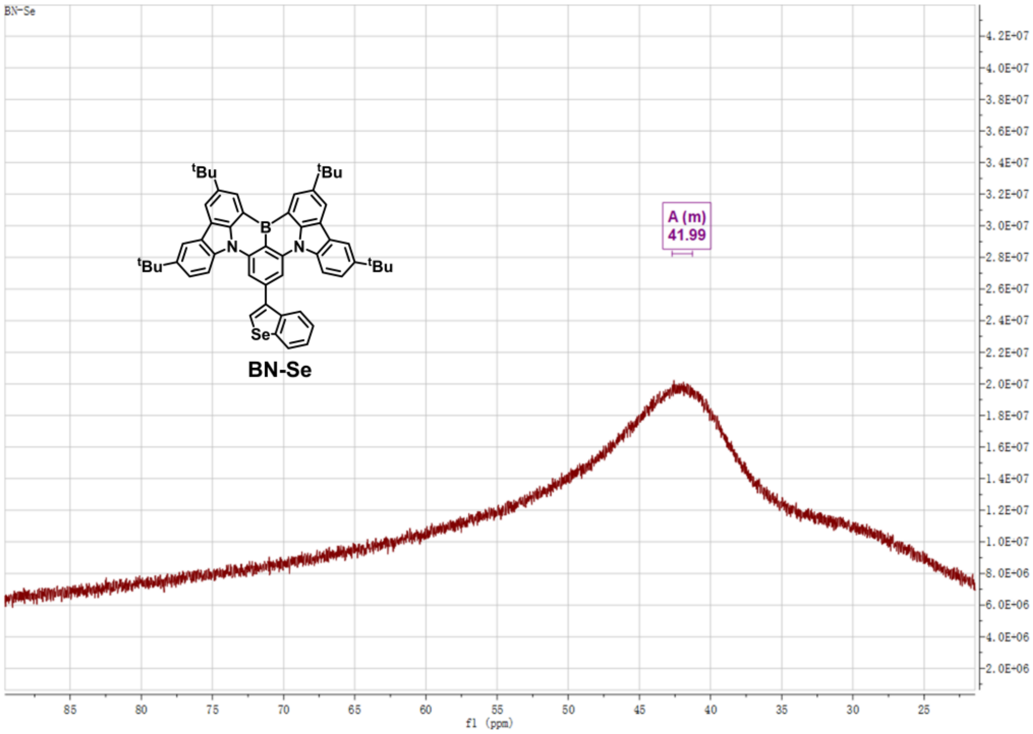

Figure S7. ^{11}B NMR spectrum of **BN-Se** measured in CD_2Cl_2.

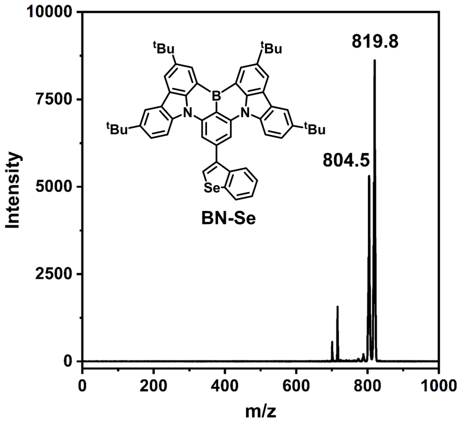

Figure S8. MALDI-TOF MS of **BN-Se**.

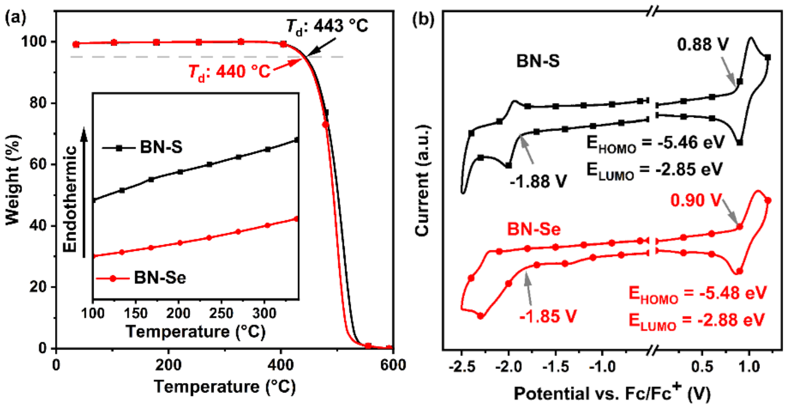

Figure S9. (a) TGA and DSC traces (inset) for **BN-S** and **BN-Se** (the decomposition temperature determined from the temperature at 5 wt% loss); (b) Cyclic voltammograms for **BN-S** and **BN-Se** in anhydrous DMF and dichloromethane with 0.1 M $TBAPF_6$ as the electrolyte for negative and positive scans, respectively, and Fc^+/Fc redox couple as the internal standard.

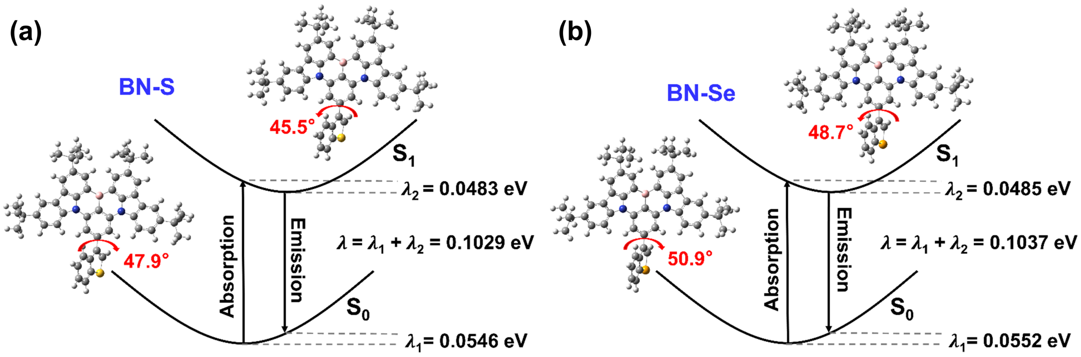

Figure S10. Optimized S_0 and S_1 structures and reorganization energies of (a) **BN-S** and (b) **BN-Se**.

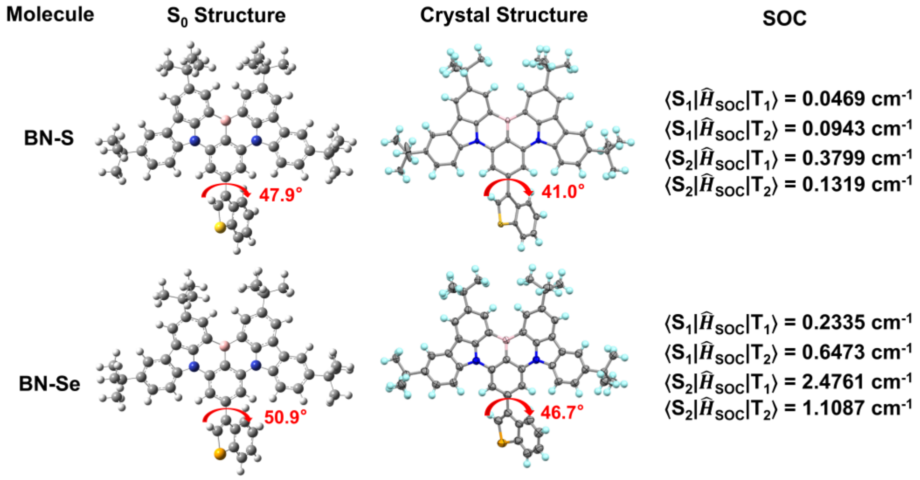

Figure S11. The optimized S_0 geometries, thermal ellipsoid plot of the single crystal structures (ellipsoids are drawn at the 50% probability level and solvent molecules have been omitted for clarity), and SOC constants of **BN-S** and **BN-Se** based on the X-ray structures.

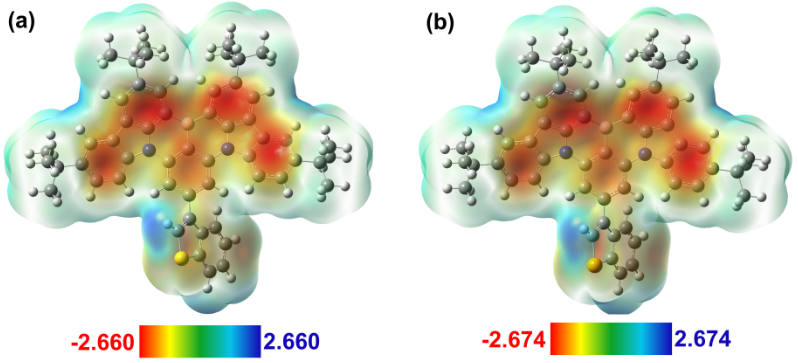

Figure S12. The molecular surface electrostatic potentials (ESP) of (a) **BN-S** and (b) **BN-Se** in their respective S_0 geometries (measuring scale × 10^{-2}, red and blue indicate negative and positive electrostatic potentials, respectively).

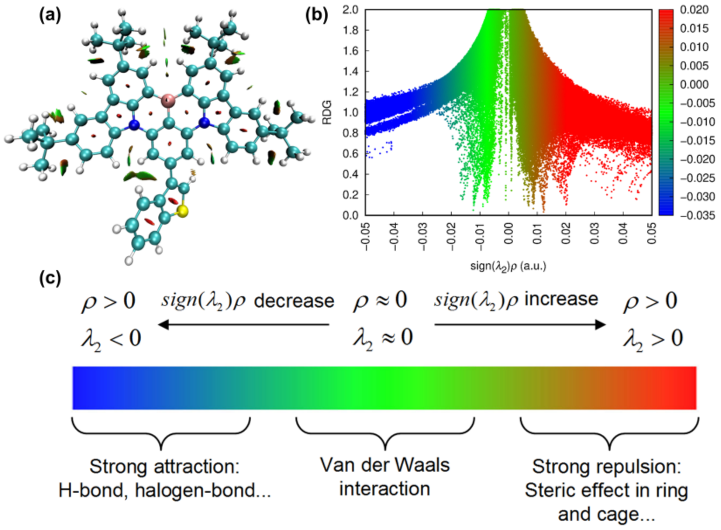

Figure S13. (a) Calculated reduced density gradient (RDG) isosurface based on the optimized S_1 geometry of **BN-S**; (b) Scattering diagram; (c) Standard coloring method and chemical explanation of sign(λ_2)ρ (the amplitude of the electron density corresponding to different types of interactions) on RDG isosurface.

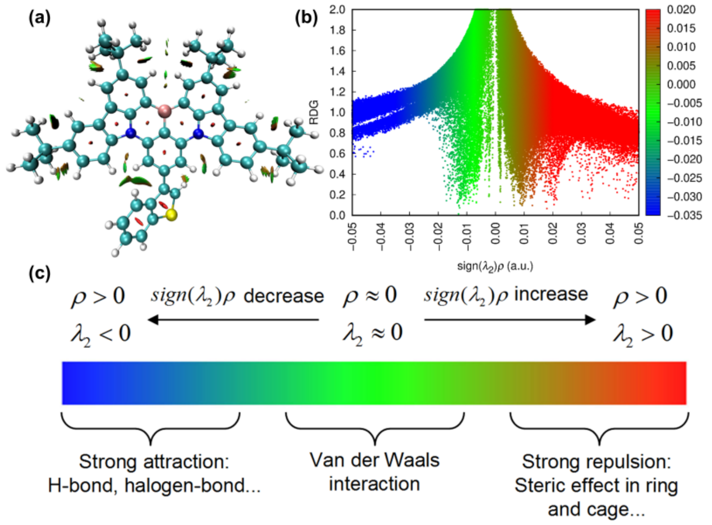

Figure S14. (a) Calculated reduced density gradient (RDG) isosurface based on the optimized S_1 geometry of **BN-Se**; (b) Scattering diagram; (c) Standard coloring method and chemical explanation of sign(λ_2)ρ (the amplitude of the electron density corresponding to different types of interactions) on RDG isosurface.

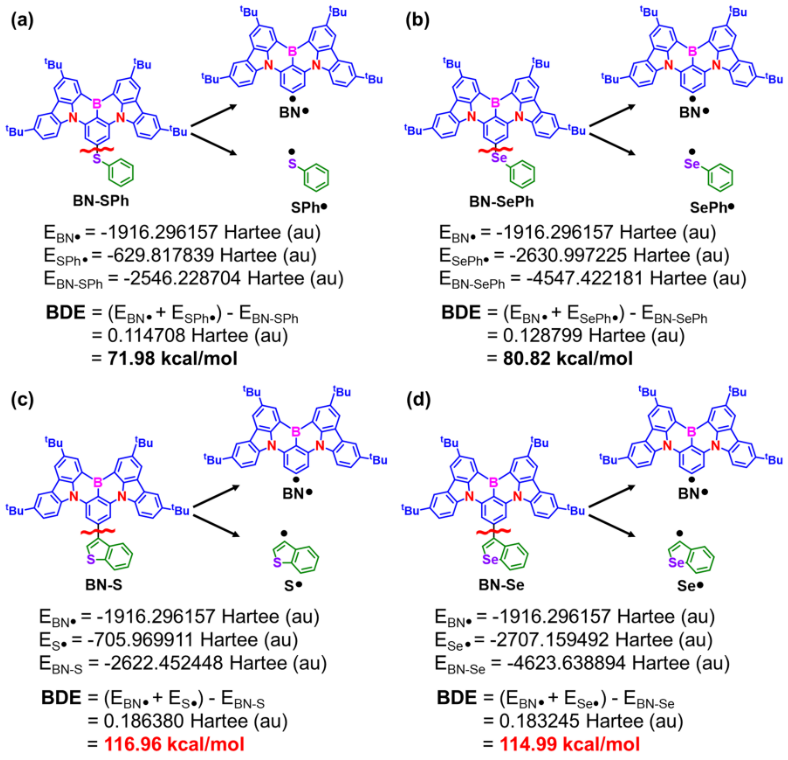

Figure S15. Bond dissociation energy (BDE) calculated based on the DFT-optimized structures of the C-S connected model compound (a) **BN-SPh**, the C-Se connected model compound (b) **BN-SePh**, the C-C connected structure of (c) **BN-S** and (d) **BN-Se**.

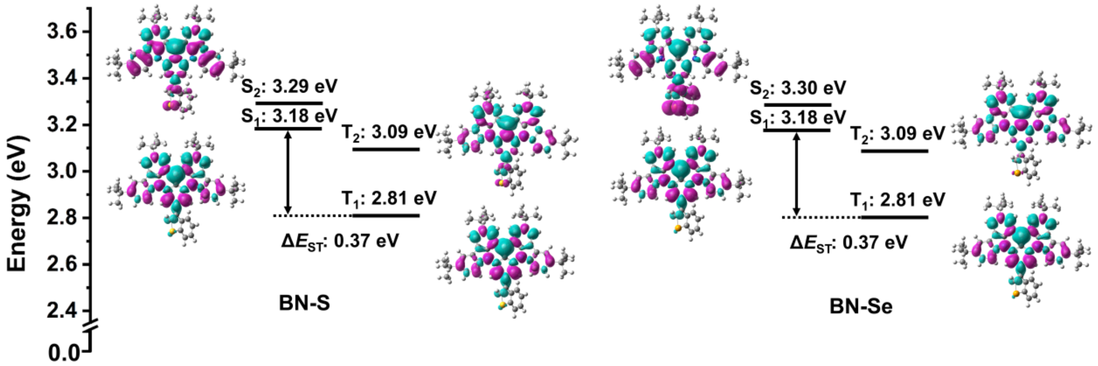

Figure S16. Simulated energy-level diagrams, NTO distributions of **BN-S** and **BN-Se**.

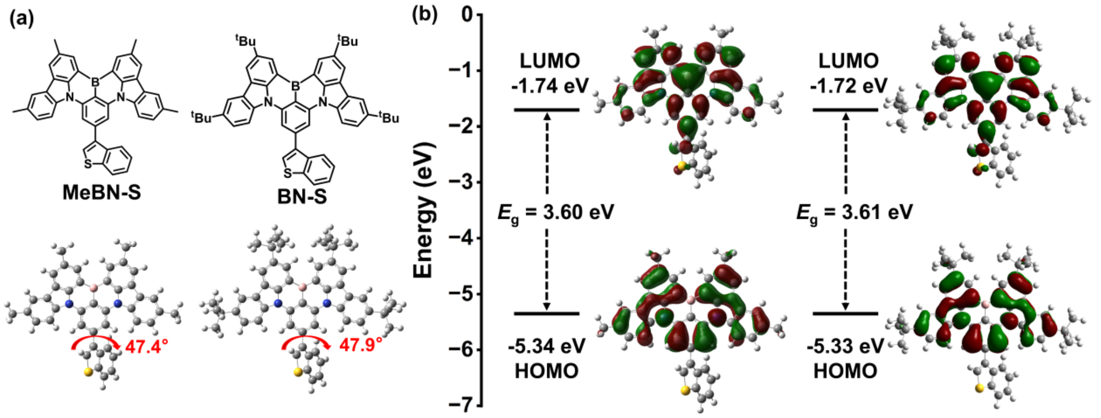

Figure S17. (a) The molecular structures and optimized S_0 geometries of the model compound **MeBN-S** and **BN-S**; (b) Calculated HOMO-LUMO energies and distribution for the model compound **MeBN-S** and **BN-S** at the PBE0/6-31G(d,p) level in the gas phase.

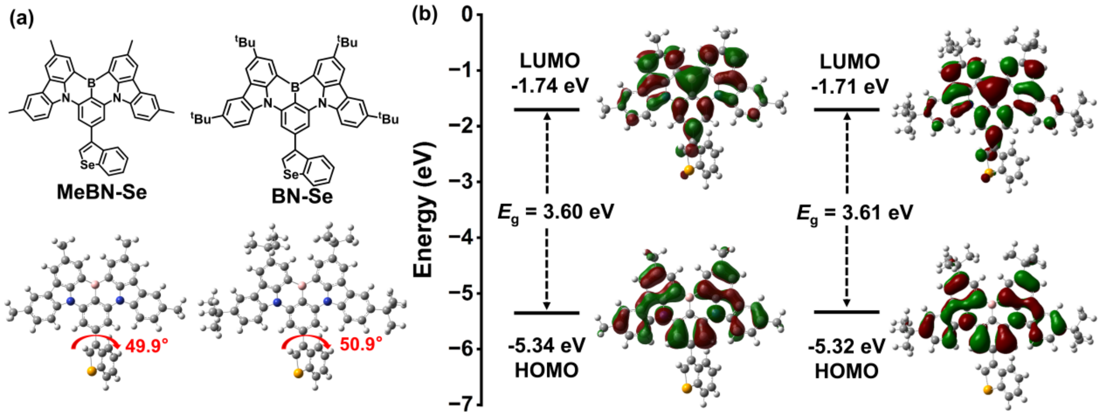

Figure S18. (a) The molecular structures and optimized S_0 geometries of the model compound **MeBN-Se** and **BN-Se**; (b) Calculated HOMO-LUMO energies and distribution for the model compound **MeBN-Se** and **BN-Se** at the PBE0/6-31G(d,p) level in the gas phase.

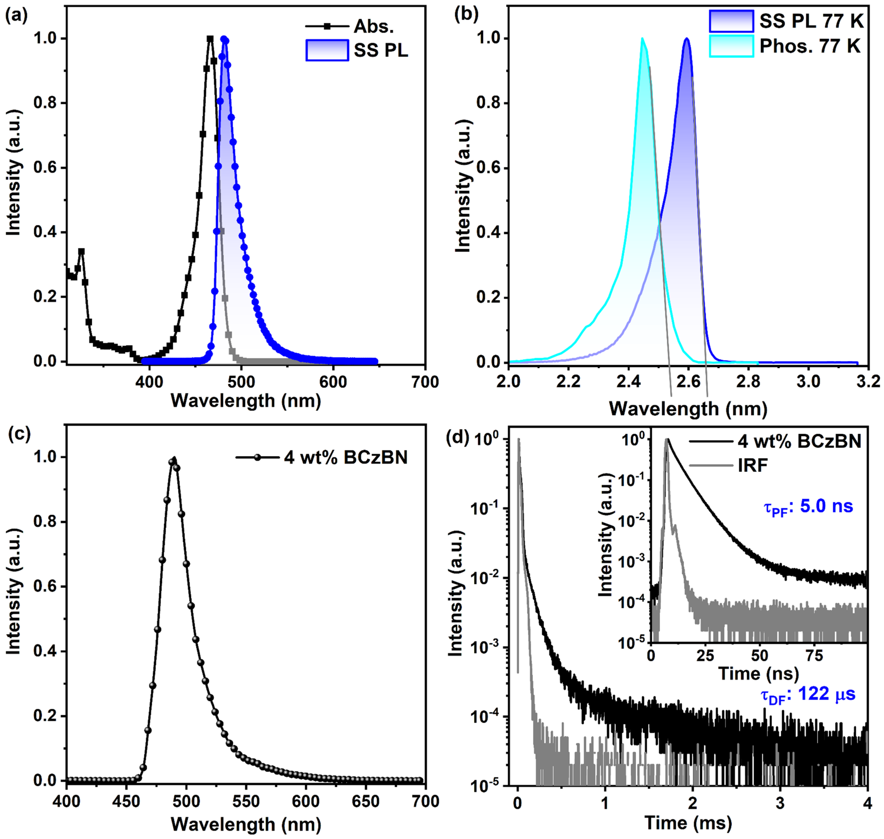

Figure S19. (a) Absorption and steady-state PL (SS-PL) spectra of **BCzBN** in dilute toluene at room temperature (λ_{exc} = 360 nm); (b) Steady-state PL (77 K) and phosphorescence spectra (1–10 ms, 77 K) of **BCzBN** in toluene (λ_{exc} = 360 nm); (c) Steady-state PL spectra and (d) Time-resolved PL decays of **BCzBN** as 4 wt% doped films in PhCzBCz (λ_{exc} = 360 nm).

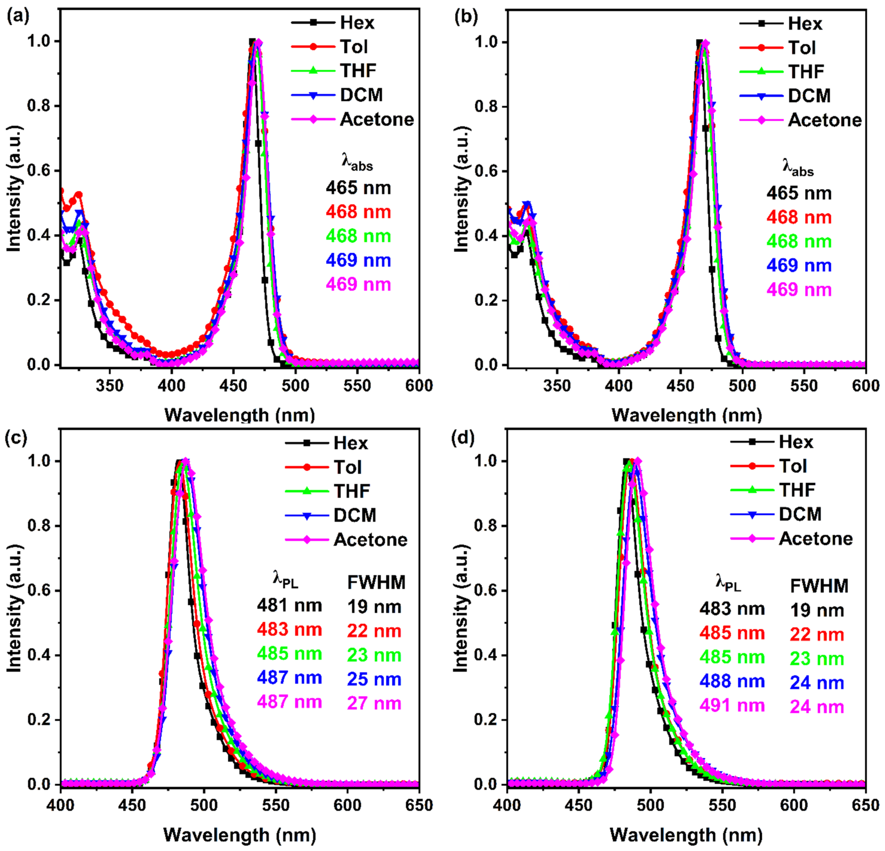

Figure S20. The absorption spectra of (a) **BN-S**, (b) **BN-Se** and PL spectra of (c) **BN-S**, (d) **BN-Se** in different polarity solvents (10^{-5} M, λ_{exc} = 360 nm).

Table S1. The absorption and emission bands, FWHM, and F_{PL} values of **BN-S** and **BN-Se** in solvents of different polarities.

	BN-S				BN-Se			
	λ_{abs} (nm)	λ_{PL} (nm)	FWHM (nm)	Φ_{PL} (%)	λ_{abs} (nm)	λ_{PL} (nm)	FWHM (nm)	Φ_{PL} (%)
hexane	465	481	19	100	465	483	19	99
toluene	468	483	22	100	468	485	22	98
THF	468	485	23	99	468	485	23	98
DCM	469	487	25	98	469	488	24	97
acetone	469	487	27	97	469	491	24	96

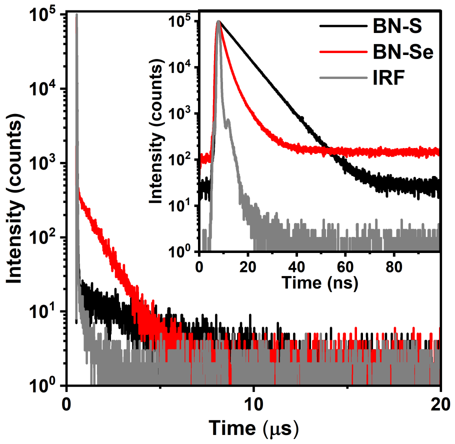

Figure S21. Time-resolved PL decays of **BN-S** and **BN-Se** in oxygen-free toluene after bubbling nitrogen for 10 min (10^{-5} M, $\lambda_{exc} = 360$ nm).

Table S2. Detailed photophysical data of **BN-S** and **BN-Se** in oxygen-free toluene after bubbling nitrogen for 10 min (10^{-5} M, $\lambda_{exc} = 360$ nm).

Emitters	Φ_{PL}[a] (%)	Φ_p[b] (%)	Φ_d[c] (%)	τ_p[d] (ns)	τ_d[e] (μs)	k_r[f] (10^7 s^{-1})	k_{nr}[g] (10^6 s^{-1})	k_{ISC}[h] (10^8 s^{-1})	k_{RISC} (10^6 s^{-1})
**BN-S**	100	30.7	69.3	6.8	2.7	4.5	0.045	1.0	1.2
**BN-Se**	98	19.0	81.0	2.7	0.7	7.0	1.4	3.0	7.6

[a] The total fluorescence quantum yield. [b] The prompt fluorescent (Φ_F) component of Φ_{PL}. [c] The delayed fluorescent (Φ_{TADF}) component of Φ_{PL}. [d] The lifetimes of prompt fluorescent (τ_F). [e] The lifetimes of TADF (τ_d). [f] The rate constants of radiative decay (k_r). [g] The rate constants of nonradiative decay (k_{nr}). [h] The rate constants of intersystem crossing (k_{ISC}). Φ_p and Φ_d are distinguished from the total Φ_{PL} by comparing the integrated intensities of prompt and delayed components in the transient PL spectra ($\lambda_{exc} = 360$ nm).[21,28,49]

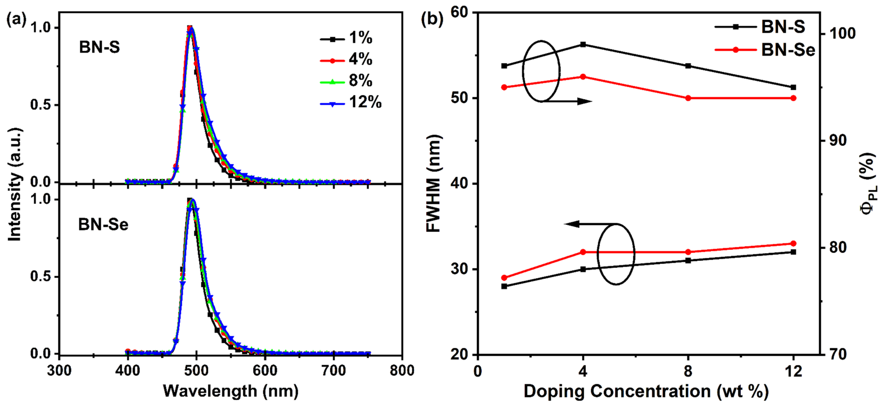

Figure S22. (a) PL spectra of doped films of **BN-S** and **BN-Se** in PhCzBCz as host at different doping concentrations; (b) Plots for the FWHM and F_{PL} versus doping concentration in doped films (λ_{exc} = 360 nm).

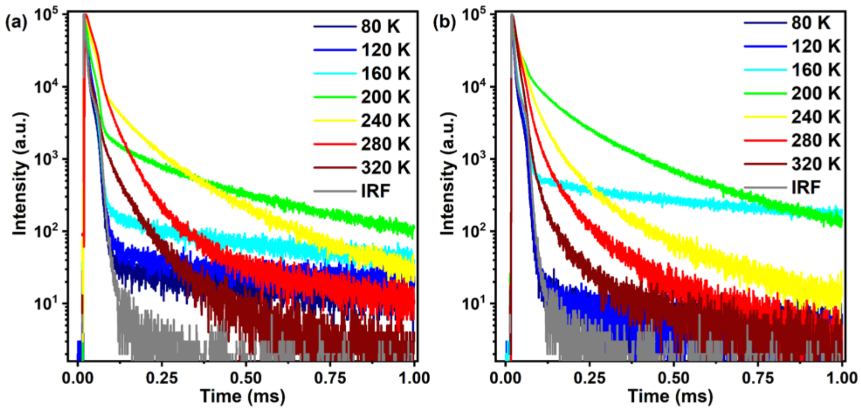

Figure S23. Variable-temperature transient PL decays of 4 wt% doped films of (a) **BN-S** and (b) **BN-Se** in PhCzBCz (λ_{exc} = 360 nm).

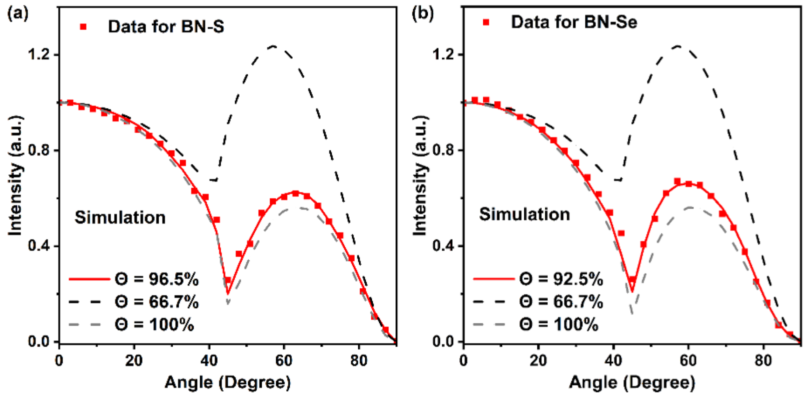

Figure S24. Variable-angle PL measurements of 4 wt% doped films of (a) **BN-S** and

(b) **BN-Se** in PhCzBCz.

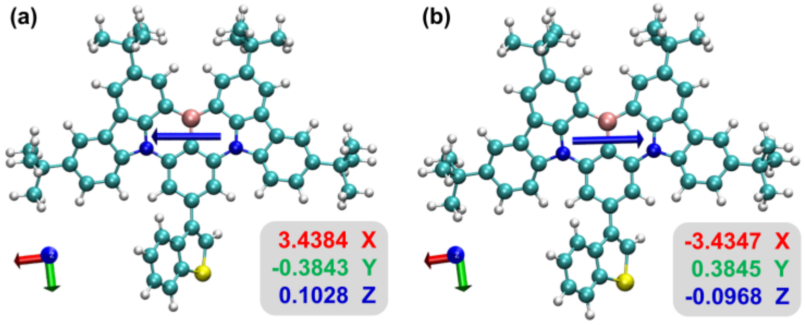

Figure S25. The calculated transition dipole moments (TDM) of (a) **BN-S** and (b) **BN-Se**.

Table S3. Detailed photophysical data of 4 wt% doped films of emitters in PhCzBCz.

Emitters	Φ_{PL}[a] (%)	Φ_{p}[b] (%)	Φ_{d}[c] (%)	τ_{p}[d] (ns)	τ_{d}[e] (μs)	k_{r}[f] (10^8 s^{-1})	k_{nr}[g] (10^6 s^{-1})	k_{ISC}[h] (10^8 s^{-1})	k_{RISC} (10^5 s^{-1})
**BCzBN**	92	52.6	39.4	5.0	122	1.1	0.9	0.86	0.14
**BN-S**	99	34.7	64.3	5.2	11.5	0.7	0.7	1.2	2.5
**BN-Se**	96	43.2	52.8	2.2	3.5	1.7	7.3	2.7	7.2

[a] The total fluorescence quantum yield. [b] The prompt fluorescent (Φ_F) component of Φ_{PL}. [c] The delayed fluorescent (Φ_{TADF}) component of Φ_{PL}. [d] The lifetimes of prompt fluorescent (τ_F). [e] The lifetimes of TADF (τ_d). [f] The rate constants of radiative decay (k_r). [g] The rate constants of nonradiative decay (k_{nr}). [h] The rate constants of intersystem crossing (k_{ISC}).

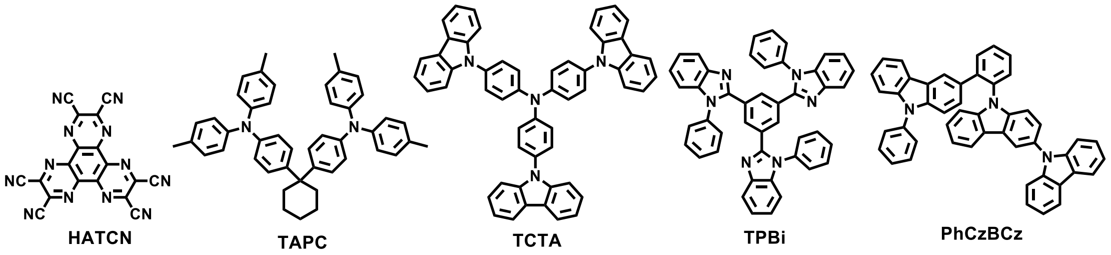

Figure S26. The chemical structures of all transport layers and host material in the doped devices.

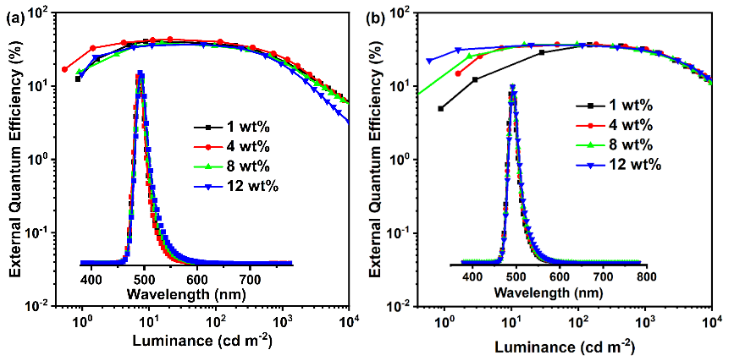

Figure S27. EQE versus luminance curves (inset: the EL spectra) of the devices with (a) **BN-S** and (b) **BN-Se** at different dopant concentrations in the EML.

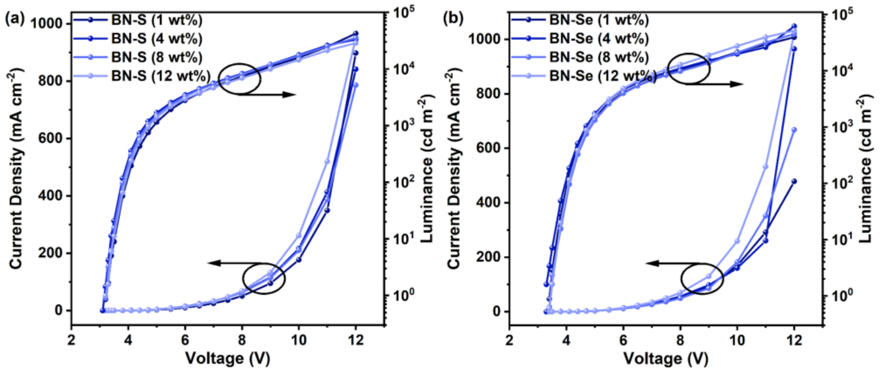

Figure S28. Luminance-voltage-current density curves of the devices with (a) **BN-S** and (b) **BN-Se** at different dopant concentrations in the EML.

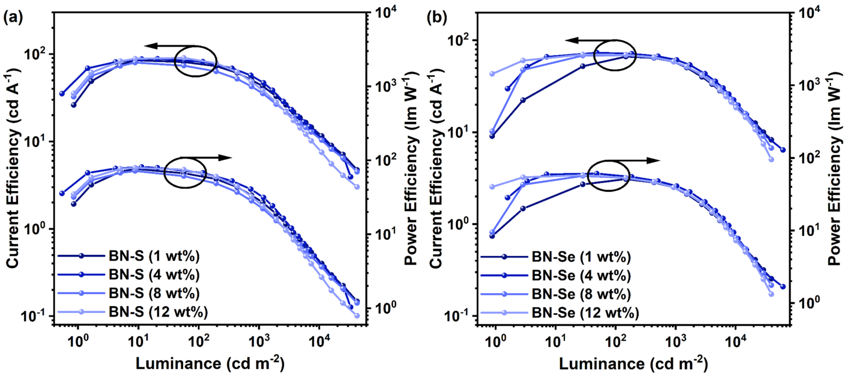

Figure S29. CE/PE versus luminance curves characteristics of the devices with (a) **BN-S** and (b) **BN-Se** at different dopant concentrations in the EML.

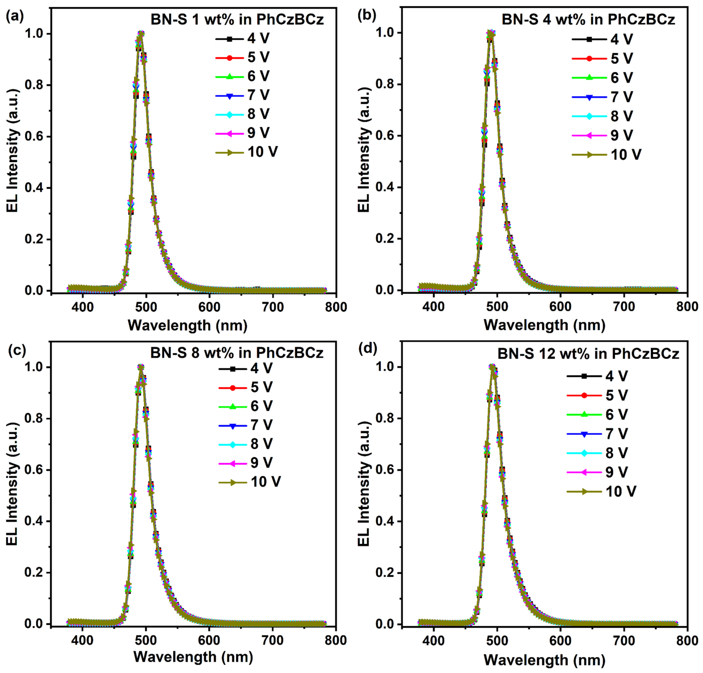

Figure S30. The EL spectra of devices with **BN-S** operated at different voltages and with various dopant concentrations (a) 1 wt%, (b) 4 wt%, (c) 8 wt%, and (d) 12 wt% in PhCzBCz.

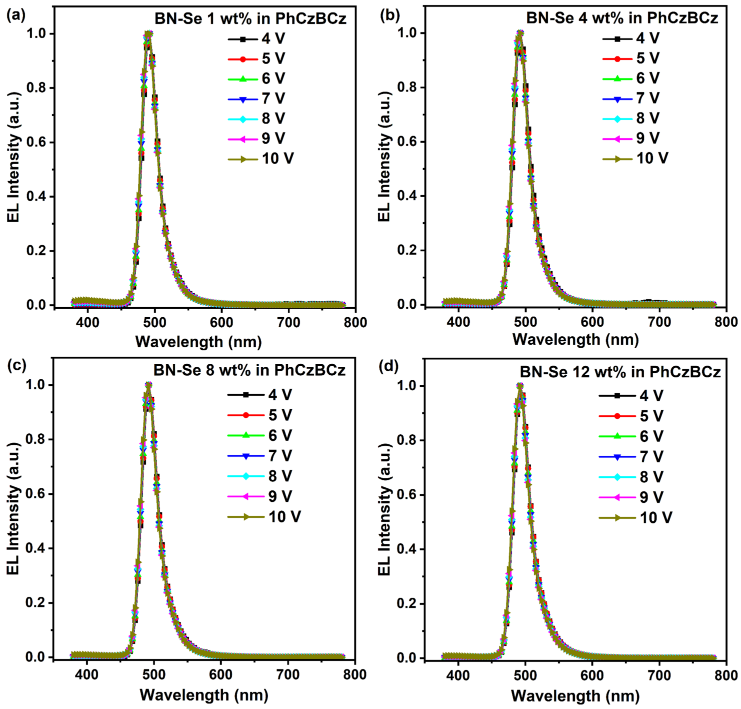

Figure S31. The EL spectra of devices with **BN-Se** operated at different voltages and with different dopant concentrations (a) 1 wt%, (b) 4 wt%, (c) 8 wt%, and (d) 12 wt% in PhCzBCz.

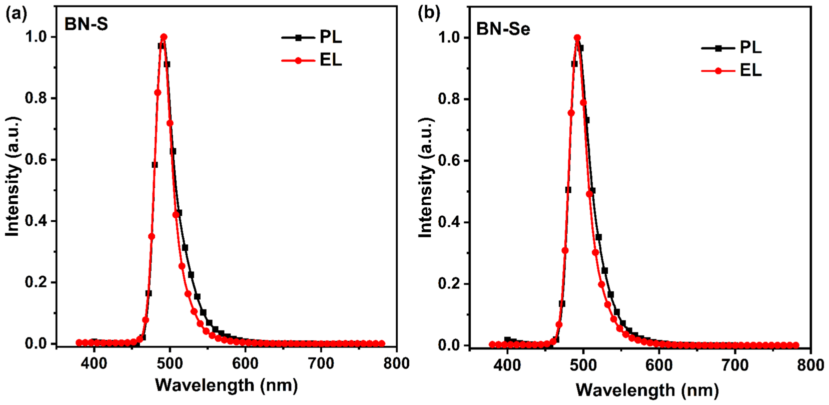

Figure S32. The PL spectra in PhCzBCz deposited films and EL spectra of (a) **BN-S** and (b) **BN-Se**-based devices at 4 wt% doping concentration.

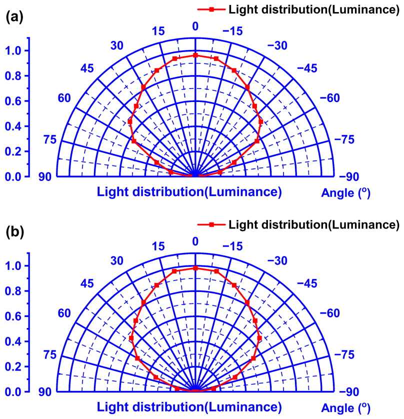

Figure S33. Angle-dependent EL intensity of the devices with (a) **BN-S** and (b) **BN-Se**. The corresponding Lambertian coefficients are calculated to be 0.96 and 0.98, respectively.

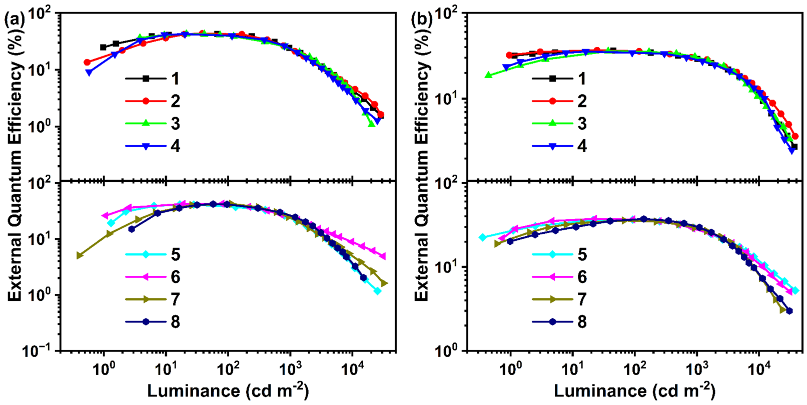

Figure S34. The EQE-luminance curves of eight groups of devices with (a) **BN-S** and (b) **BN-Se** at 4 wt% doping concentration.

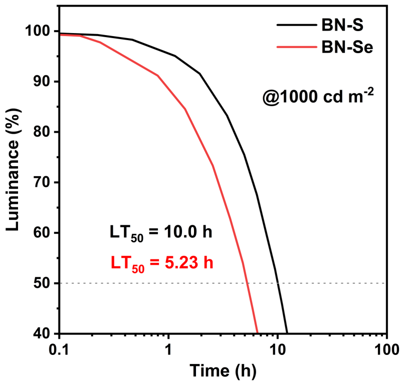

Figure S35. Luminance versus time of the devices with **BN-S** and **BN-Se** at an initial luminance of 1000 cd m^{-2}.

Table S4. Performance summary of mono-boron MR-TADF OLEDs with emission peak between 470–540 nm.

Devices	λ_{EL} [nm]	V_{on} [V]	CE_{max} [cd A^{-1}]	PE_{max} [lm W^{-1}]	EQE[a] [%]	FWHM [nm]	CIE [x, y]	Ref
**BN-S**	492	3.1	88.3	81.1	43.1/40.2/24.8	27	(0.09, 0.42)	This work
**BN-Se**	492	3.3	73.4	60.7	36.9/36.7/31.8	28	(0.09, 0.43)	This work
IDAD-BNCz	498	3.1	90.5	82.7	34.3/31.8/14.0	34	(0.12, 0.51)	13
PSeZBN2	517	2.5	104.9	109.7	29.5/-/29.0	57	–	14
BN-Ad	500	3.5	59.6	48.0	32.3/22.3/13.3	35	(0.14, 0.57)	15
o-BNPO	496	-	73.9	68.3	36.0/29.9/14.8	26	(0.09,0.48)	16
m-PAz-BNCz	528	2.7	135.3	152.1	35.7/34.4/25.3	39	(0.28, 0.68)	17
SetBuNBN	483	3.7	36.5	30.2	25.4/24.4/19.6	25	–	18
D-dm-ICz-BNCz	534	3.7	-	105.8	32.9/25.0/17.7	46	(0.30,0.67)	19
BNCPPt	507	3.3	41	32.5	13.5/13.0/12.9	35	(0.22,0.63)	20
TRZCzPh-BNCz	513	3.2	-	101.4	32.5/30.5/22.4	37	(0.17,0.68)	21
D-TCz-VTCzBN	524	3.8	129.3	96.7	32.2/18.0/16.0	37	(0.22,0.71)	22
BN-Se	506	2.4	95.9	103.9	32.6/-/32.2	45	(0.15,0.62)	23
DCz-BSeN	481	3.5	-	-	22.3/19.6/15.6	32	(0.11, 0.25)	24
D-p−1-PCzBN	496	3.9	71.4	51.0	33.9/26.5/16.3	26	(0.08, 0.52)	25
TPh-BN	492	3.7	54.8	43.1	28.9/25.1/15.6	28	(0.10,0.46)	26
TCzBN-SO	512	-	106.3	111.2	33.0/27.7/11.6	36	(0.17, 0.68)	27
BNCz-pTPA	496	3.6	67.2	58.7	27.3/22.3/13.3	34	(0.12,0.54)	28
DCzBN–Au	510	-	112.5	106.8	35.8/35.7/32.3	34	(0.16.0.67)	29
BN-STO	517	2.41	141.2	176.9	40.1/39.0/28.1	34	(0.19,0.70)	30
(BzIPr)AuBN	510	2.5	97.1	77.8	30.3/28.1/22.2	34	(0.16, 0.68)	31
D-Spiro-BNCz	533	4.2	140.5	90.1	34.2/22.5/17.2	48	(0.29, 0.67)	32
tPhCzPh3Si	512	3.6	112.3	80.1	34.6/26.1/16.1	28	(0.14,0.70)	33
(P/M)-BN-P	532	3.1	119.6	118.9	30.6/23.7/10.0	37	(0.29, 0.68)	34
BN-TP	528	3.1	139.3	139.3	35.1/32.4/20.8	36	(0.26, 0.70)	35
D-Cz-BN	489	3.1	-	-	28.7/22.6/11.4	24	(0.10, 0.41)	36
DtCzB-TPTRZ	520	2.8	105.8	116.2	30.6/28.6/16.4	41	(0.23, 0.68)	37
BN-CP1	496	2.4	83.8	109.7	40.0/34.0/18.5	25	(0.09, 0.50)	38
Se-SFBN	488	4.0	60.0	47.7	35.6/30.3/22.1	27	-	39
BNCz-NPO	480	3.0	43.0	45.0	32.1/21.7/9.7	26	(0.11, 0.22)	40
pPXSe-BN	496	2.6	82.8	100.0	37.1/30.2/20.4	29	(0.10, 0.50)	41
BN-PCz-TPA	496	2.9	84.4	87.9	36.3/31.8/13.8	27	(0.08, 0.51)	42
m-DCzDAz-BNCz	512	2.9	106.9	114.4	34.8/31.4/27.2	35	(0.17, 0.65)	43
BN-TP-N3	524	3.0	135.3	139.9	37.3/34.7/19.8	33	(0.23, 0.71)	44
TPA-CN-BN	504	3.0	102.5	94.7	37.9/34.8/21.8	31	(0.18, 0.62)	45

2GtBuCzCO$_2$HDCzB	495	-	61.2	35.0	27.9/27.6/22.3	27	(0.08, 0.53)	46
tCzBT1B	495	3.5	79.1	66.7	34.9/28.1/18.0	33	(0.14, 0.59)	47
m-PAz-BNCz	528	3.0	132.1	133.9	36.2/30.8/19.5	37	(0.26, 0.70)	48
BN-PhAzCz	528	2.9	148.5	159.0	38.2/36.5/27.5	39	(0.26, 0.69)	49
AB2Me	488	3.1	60.4	59.3	31.1/27.7/9.6	22	(0.10, 0.39)	50

[a]EL emission peak; [b]Turn-on voltage; [c]Maximum current efficiency; [d]Maximum power efficiency; [e]External quantum efficiency of maximum/at 100 cd m^{-2}/1000 cd m^{-2}.

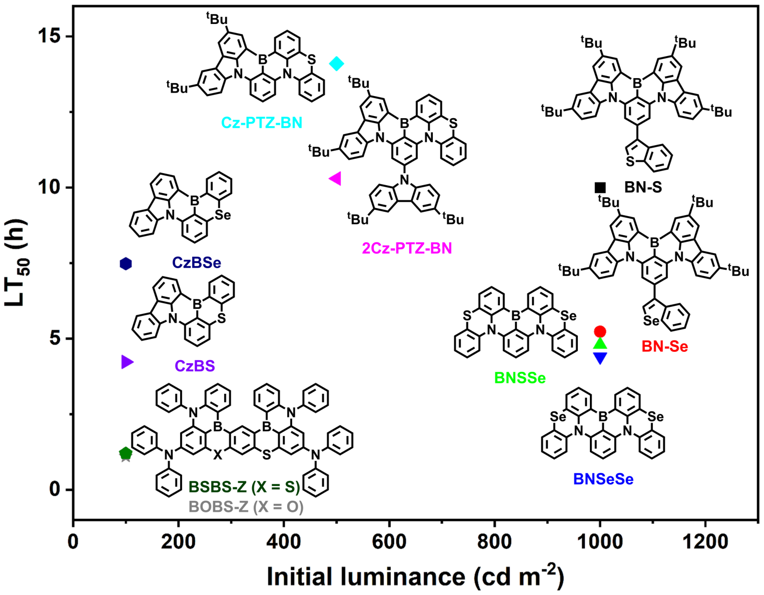

Figure S36. Lifetimes (LT_{50}) versus initial luminance of other sulfur- and selenium-containing MR-TADF OLEDs (inset: the molecular structures).

Table S5. Lifetime performance summary of other sulfur- and selenium-containing MR-TADF OLEDs.

Emitters	Initial luminance [cd m^{-2}]	Lifetimes LT_{50} [h]	Ref
BN-S	1000	10.0	This work
BN-Se	1000	5.23	This work
BNSSe	1000	4.8	51
BNSeSe	1000	4.4	51
Cz-PTZ-BN	500	14.1	52
2Cz-PTZ-BN	500	10.3	52
CzBS	100	4.23	53
CzBSe	100	7.48	53
BOBS-Z	100	1.1	54
BSBS-Z	100	1.2	54

3. References

1 N. O. C. Winter, C. Hättig, *J. Chem. Phys.* **2011**, *134*, 184101.

2 S. Hirata, M. Head-Gordon, *Chem. Phys. Lett.* **1999**, *314*, 291-299.

3 C. Adamo, V. Barone, *J. Chem. Phys.* **1999**, *110*, 6158-6170.

4 G. A. Petersson, M. A. Al-Laham, *J. Chem. Phys.* **1991**, *94*, 6081-6090.

5 E. Runge, E. K. U. Gross, *Phys. Rev. Lett.* **1984**, *52*, 997-1000.

6 K. B. Wiberg, *J. Comput. Chem.* **2004**, *25*, 1342-1346.

7 C. Hättig, K. Hald, Phys. *Chem. Chem. Phys.* **2002**, *4*, 2111-2118.

8 O. S. Lee, M. Gather, E. Zysman-Colman, *Digit. Discov.* ***2024**, 3, 1695*

9 X. Gao, S. Bai, D. Fazzi, T. Niehaus, M. Barbatti, W. Thiel, *J. Chem. Theory Comput.* **2017**, *13*, 515.

10 T. Lu, F. Chen, *J. Comput. Chem.* **2012**, *33*, 580-592.

11 Humphrey, W., Dalke, A.; Schulten, K. VMD: visual molecular dynamics. *J. Mol.Graph.* **1996**, *14*, 33-38.

12 S. M. Chintala, J. C. Throgmorton, P. F. Maness, R. D. McCulla, *J. Phys. Org. Chem.* **2021**, *34*, e4144.

13 J. M. Jin, D. Liu, W. C. Chen, C. Shi, G. Chen, X. Wang, L. Xing, W. Ying, S. Ji, Y. Huo, S. J. Su, *Angew. Chem. Int. Ed.* **2024**, *63*, e202401120.

14 Y. Zou, M. Yu, Y. Xu, Z. Xiao, X. Song, Y. Hu, Z. Xu, C. Zhong, J. He, X. Cao, K. Li, J. Miao, C. Yang, *Chem* **2024**, *10*, 1485-1501.

15 Y. Qi, Z. Zhang, W. Sun, S. Wu, J. Liu, Z. Lin, P. Jiang, H. Yu, L. Zhou, G. Lu,

J. Mater. Chem. C **2024**, *12*, 6319-6325.

16 X. Xiong, J. Q. Li, T. F. Chen, X. C. Fan, Y. C. Cheng, H. Wang, F. Huang, H. Wu, J. Yu, X. K. Chen, K. Wang, X. H. Zhang, *Adv. Funct. Mater.* **2024**, *34*, 2313726.

17 Q. Wang, T. Huang, Y. Qu, X. Song, Y. Xu, Y. Wang, *ACS Appl. Mater. Interfaces* **2024**, *16*, 4948-4957.

18 J. Jin, S. Wang, H. Jiang, L. Wang, W.-Y. Wong, *Adv. Opt. Mater.* **2024**, *12*, 2302354.

19 S. Q. Song, C. F. Yip, Q. M. Liu, X. S. Zhong, Y. Wang, Y. X. Zheng, *Adv. Opt. Mater.* **2024**, *12*, 2400200.

20 Y. Feng, X. Zhuang, Y. Xu, J. Xue, C. Qu, Q. Wang, Y. Liu, Y. Wang, *Chem. Eng. J.* **2023**, *478*, 147123.

21 Y. Liu, X. Xiao, Z. Huang, D. Yang, D. Ma, J. Liu, B. Lei, Z. Bin, J. You, *Angew. Chem. Int. Ed.* **2022**, *61*, e202210210.

22 X. F. Luo, S. Q. Song, H. X. Ni, H. Ma, D. Yang, D. Ma, Y. X. Zheng, J. L. Zuo, *Angew. Chem. Int. Ed.* **2022**, *61*, e202209984.

23 X. Cao, K. Pan, J. Miao, X. Lv, Z. Huang, F. Ni, X. Yin, Y. Wei, C. Yang, *J. Am. Chem. Soc.* **2022**, *144*, 22976-22984.

24 Q. Li, Y. Wu, Q. Yang, S. Wang, S. Shao, L. Wang, *ACS Appl. Mater. Interfaces* **2022**, *14*, 49995-50003.

25 X. F. Luo, H. X. Ni, X. Liang, D. Yang, D. Ma, Y. X. Zheng, J. L. Zuo, *Adv. Opt. Mater.* **2023**, *11*, 2203002.

26 F. Liu, Z. Cheng, L. Wan, Z. Feng, H. Liu, H. Jin, L. Gao, P. Lu, W. Yang, *Small* **2022**, *18*, 2106462.

27 F. Huang, Y.-C. Cheng, H. Wu, X. Xiong, J. Yu, X.-C. Fan, K. Wang, X.-H. Zhang, *Chem. Eng. J.* **2023**, *465*, 142900.

28 G. Chen, J. Wang, W. C. Chen, Y. Gong, N. Zhuang, H. Liang, L. Xing, Y. Liu, S. Ji, H. L. Zhang, Z. Zhao, Y. Huo, B. Z. Tang, *Adv. Funct. Mater.* **2023**, *33*, 2211893.

29 J. Wang, N. Li, C. Zhong, J. Miao, Z. Huang, M. Yu, Y. X. Hu, S. Luo, Y. Zou, K. Li, C. Yang, *Adv. Mater.* **2022**, *35*, 2208378.

30 Y. Hu, J. Miao, C. Zhong, Y. Zeng, S. Gong, X. Cao, X. Zhou, Y. Gu, C. Yang, *Angew. Chem. Int. Ed.* **2023**, *62*, e202302478.

31 S. Cai, G. S. M. Tong, L. Du, G. K. M. So, F. F. Hung, T. L. Lam, G. Cheng, H. Xiao, X. Chang, Z. X. Xu, C. M. Che, *Angew. Chem. Int. Ed.* **2022**, *61*, e202213392.

32 X. F. Luo, S. Q. Song, X. Wu, C. F. Yip, S. Cai, Y. X. Zheng, *Aggregate* **2023**, *5*, e445.

33 H.-X. Ni, W. Sun, X.-F. Luo, L. Yuan, X. Liang, X.-J. Liao, L. Zhou, Y.-X. Zheng, *The Innovation Mater.* **2023**, *1*, 100041.

34 Q. Wang, L. Yuan, C. Qu, T. Huang, X. Song, Y. Xu, Y. X. Zheng, Y. Wang, *Adv. Mater.* **2023**, *35*, 2305125.

35 Y. Xu, Q. Wang, J. Wei, X. Peng, J. Xue, Z. Wang, S. J. Su, Y. Wang, *Angew. Chem. Int. Ed.* **2022**, *61*, e202204652.

36 Y. Zhang, J. Wei, D. Zhang, C. Yin, G. Li, Z. Liu, X. Jia, J. Qiao, L. Duan, *Angew. Chem. Int. Ed.* **2022**, *61*, e202113206.

37 Y. Xu, C. Li, Z. Li, J. Wang, J. Xue, Q. Wang, X. Cai, Y. Wang, *CCS Chem.* **2022**, *4*, 2065-2079.

38 P. Jiang, J. Miao, X. Cao, H. Xia, K. Pan, T. Hua, X. Lv, Z. Huang, Y. Zou, C. Yang, *Adv. Mater.* **2022**, *34*, 2106954.

39 Q. Zheng, Y.-K. Qu, P. Zuo, H.-T. Yuan, Y.-J. Yang, Y.-C. Qiu, L.-S. Liao, D.-Y. Zhou, Z.-Q. Jiang, *Chem* **2025**, *11*, 102353.

40 L. Xing, J. Wang, W.-C. Chen, B. Liu, G. Chen, X. Wang, J.-H. Tan, S. S. Chen, J.-X. Chen, S. Ji, Z. Zhao, M.-C. Tang, Y. Huo, *Nat. Commun.* **2024**, *15*, 6175.

41 Z. Chen, D. Liu, M. Li, Y. Jiao, Z. Yang, K. Liu, S. J. Su, *Adv. Funct. Mater.* **2024**, *34*, 2404278.

42 Y. Feng, Y. Xu, C. Qu, Q. Wang, K. Ye, Y. Liu, Y. Wang, *Adv. Mater.* **2024**, *36*, 2403061.

43 T. Huang, Y. Xu, X. Lu, Y. Qu, J. Wei, Y. Wang, *Angew. Chem. Int. Ed.* **2024**, *63*, e202411268.

44 Q. Wang, Y. Xu, T. Huang, Y. Qu, J. Xue, B. Liang, Y. Wang, *Angew. Chem. Int. Ed.* **2023**, *62*, e202301930.

45 F. Liu, Z. Cheng, W. Dong, Y. Yan, Y. Xu, Z. Su, Y. Hu, L. Wan, P. Lu, *Angew. Chem. Int. Ed.* **2025,** *64*, e202416154.

46 S. Wu, D. Chen, X.-H. Zhang, D. Sun, E. Zysman-Colman, *Adv. Mater.* **2025**, *37*, 2415289.

47 D. Chen, H. Wang, F. Huang, D. B. Cordes, A. P. McKay, K. Wang, X.-H. Zhang, E. Zysman-Colman, *Adv. Funct. Mater.* **2025**, 10.1002/adfm.202506189.

48 T. Huang, Y. Xu, Y. Qu, X. Lu, K. Ye, X. Zhuang, Y. Wang, *Adv. Mater.* **2025**, 10.1002/adma.202503383.

49 Y. Qu, Y. Xu, T. Huang, X. Song, K. Ye, Y. Wang, *Angew. Chem. Int. Ed.* **2025**, 10.1002/anie.202506201.

50 G. Chen, S. W. Chen, Y. Su, R. J. Wang, L. Huang, X. L. Liu, L. J. Xing, B. Liu, J. M. Jin, Y. Huo, S. S. Chen, S. Ji, M. C. Tang, W. C. Chen, *Adv. Funct. Mater.* **2025**, 10.1002/adfm.202501039.

51 Y. X. Hu, J. Miao, T. Hua, Z. Huang, Y. Qi, Y. Zou, Y. Qiu, H. Xia, H. Liu, X. Cao, C. Yang, *Nat. Photonics* **2022**, *16*, 803-810.

52 F. Liu, Z. Cheng, Y. Jiang, L. Gao, H. Liu, H. Liu, Z. Feng, P. Lu, W. Yang, *Angew. Chem. Int. Ed.* **2022**, *61*, e202116927.

53 I. S. Park, H. Min, T. Yasuda, *Angew. Chem. Int. Ed.* **2022**, *61,* e202205684.

54 I. S. Park, M. Yang, H. Shibata, N. Amanokura, T. Yasuda, *Adv. Mater.* **2022**, *34,* 2107951.
